# Virtual screening of *Kocuria oceani* AT-1 metabolites as potential maize growth regulators under drought conditions using molecular docking and dynamics simulation

**DOI:** 10.1371/journal.pone.0354805

**Published:** 2026-07-24

**Authors:** Muhammad Usama, Mahwish Salman, Ghulam Mustafa

**Affiliations:** Department of Biochemistry, Government College University Faisalabad, Faisalabad Pakistan; University of Sahiwal, PAKISTAN

## Abstract

Maize is a vital cereal crop, severely affected by environmental factors and climate change. Among abiotic factors, drought stress is considered one of the most detrimental factors limiting plant growth and biomass production. However, the molecular basis of bacterial metabolite-mediated drought tolerance in cereal crops remains poorly understood. The current *in silico* study aimed to investigate the molecular interactions between *Kocuria oceani* AT-1 metabolites and the maize UGT706F8 protein, using molecular docking and dynamics simulation analyses. The molecular interactions between bacterial metabolites and the UGT706F8 protein (glycosyltransferase enzyme, PDB ID 7Q3S) from *Zea mays* L. were evaluated. The *in silico* docking findings revealed a favorable binding affinity between bacterial metabolites and the target protein, indicating stable molecular interactions at the protein active site. Specifically, 4-tetradecanoyl-2,6-piperazinedione, and 3-benzylhexahydropyrrolo(1,2-a)pyrazine-1,4-dione displayed binding energies of −7.5 ± 0.33 and −8.5 ± 0.36 kcal/mol against the target protein, respectively. Under identical docking parameters, the known plant growth regulators (indole acetic acid and abscisic acid) yielded binding energies of −6.6 ± 0.34 and −5.8 ± 0.32 kcal/mol, respectively. Re-docking of the native uridine-5-diphosphate (UDP) ligand yielded a Root Mean Square Deviation (RMSD) of 0.9 Å, validating the reliability of the docking procedure. Additionally, molecular dynamics simulation (200 nanoseconds) outcomes supported these predictions, indicating stable molecular interactions and structural integrity of ligand-protein complexes, with ligand RMSD values remaining between 2 and 3 Å. The formation of hydrogen bonding (an average of 2.4 ± 0.6 and 2.1 ± 0.5, respectively), hydrophobic interactions, and stable Solvent-Accessible Surface Area (SASA) profiles (ranging between 19,000–22,000 Å^2^ and 19,000–21,000 Å^2^, respectively) suggest the stability of complexes. Moreover, Molecular Mechanics Generalized Born Surface Area (MM-GBSA) outcomes (−102.05 ± 13.96 and −54.86 ± 7.93 kcal/mol, respectively) supported the favorable binding energetics of ligand-protein complexes. These computational outcomes suggest that *Kocuria oceani* metabolites can establish stable molecular interactions with the UGT706F8 protein, which may influence glycosyltransferase function. Further *in vivo* experimental validation is required to confirm their predicted maize growth-promoting potential under drought conditions.

## Introduction

*Zea mays* L. (maize) is a widely cultivated cereal crop around the globe, serving as a staple food for humans and animals, and is also employed for biofuel production [1]. It contributes significantly to fulfilling the ever-growing demand for food. Currently, the environment is becoming more unpredictable due to climate change [2]. Among abiotic factors, drought stress is considered one of the most detrimental factors, affecting maize growth more critically in a large geographical area [3]. Prolonged water shortage limits plant growth by reducing the photosynthesis rate, disturbing the plant respiration rate, and decreasing the nutrient supply [4].

In modern agriculture, several approaches have been employed to alleviate the drought effect on plants, but they still face some restrictions [2]. Plant growth-promoting bacteria (PGPB) are known for their plant growth-promoting potential and have emerged as one of the most viable, economical, and environmentally friendly approach to boost maize resilience to drought stress [5]. Actinomycetes represent a diverse group of actinobacteria known for their potential to produce a broad spectrum of bioactive metabolites and are gaining interest as bio-priming agents [6]. However, the potential of *Kocuria oceani*, a *non-streptomyces* actinobacterium, to enhance drought resilience in cereal crops, specifically in maize, has not been investigated yet [7,8].

Some bacterial metabolites could potentially work as signaling molecules, mimicking plant hormones, i.e., IAA (indole-3-acetic acid), GA (gibberellic acid), and ABA (abscisic acid) to modulate plant physiological and biochemical attributes [9]. Furthermore, they may strengthen the antioxidant defense system and stimulate osmolyte accumulation to protect macromolecules such as proteins by capturing reactive oxygen species, which are produced as a result of drought stress [10]. Consequently, bacterial metabolites could play a promising role in plant adaptation to unpropitious environmental events [5].

Computational biology has emerged as a powerful approach to elucidate plant-microbe interactions. Molecular docking and molecular dynamics simulation emerged as promising tools that allow researchers to analyze molecular interactions between bioactive compounds and cellular proteins [11]. These approaches have revealed the structural and molecular basis of macromolecules at the atomic level and assist researchers in optimizing small molecules to regulate the functions of macromolecules before conducting *in vivo* experiments [12].

**Fig 1:**
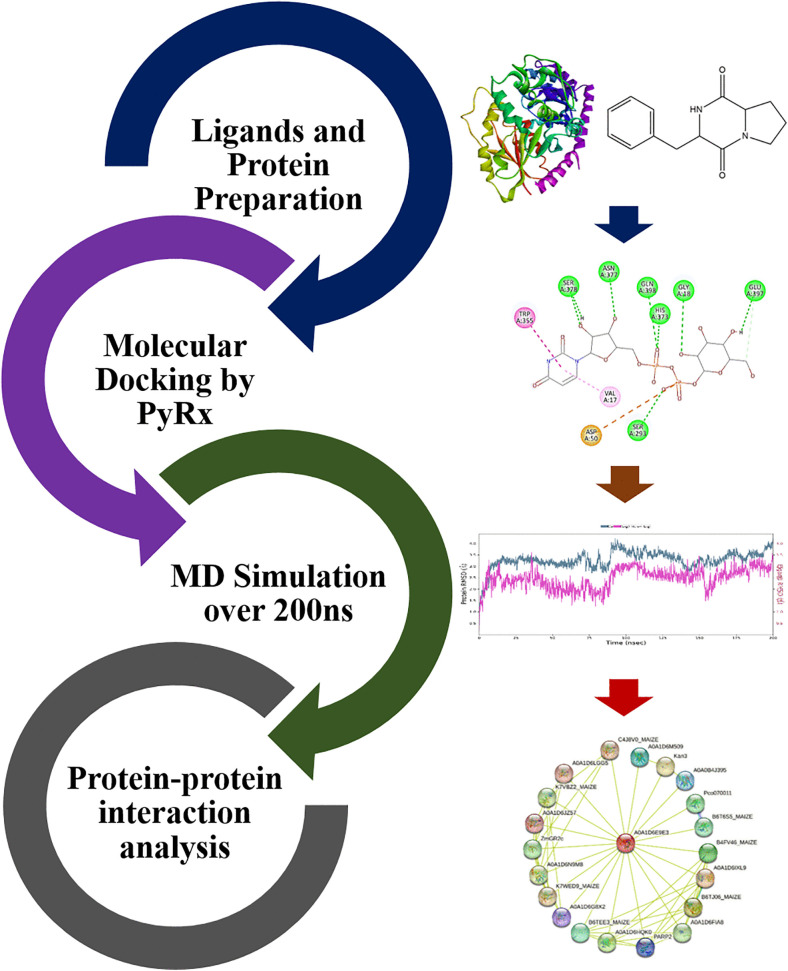
Schematic representation of the computational methodology followed in the current investigation, including molecular docking, molecular dynamics simulation, protein-protein interaction (PPI) network analysis of the target protein, and physicochemical, solubility and toxicity profiling of selected bacterial metabolites.

Secondary metabolites such as flavonoids are essential for plant stress adaptation, contributing to osmotic regulation, antioxidant defense, and signaling under drought conditions [9,10]. Although the direct role of UGT706F8 in maize drought tolerance has not yet been experimentally established, members of the Family 1 UDP-glycosyltransferase family have been implicated in abiotic stress adaptation through glycosylation of phytohormones and secondary metabolites. The computational novelty of this study lies in providing the first *in silico* investigation of *K. oceani*-derived metabolites targeting the maize glycosyltransferase enzyme (UGT706F8). Unlike previous studies, our study integrates molecular docking, molecular dynamics simulations, and binding free-energy calculations to systematically evaluate the molecular interactions and stability of metabolite-protein complexes. To the best of our knowledge, no previous study has combined these computational approaches to investigate the interaction between *K. oceani* metabolites and the UGT706F8 protein, making this study computationally novel and providing new hypotheses for future biological investigation. These outcomes may provide a pathway to develop metabolite-inspired bioactive molecules as potential growth regulators to replace conventional agrochemicals to support sustainable climate-smart agricultural practices. The step-wise methodology employed in the current study is displayed in Fig 1.

## Materials and methods

### Ligand structure retrieval

The 3D conformers of 52 *Kocuria oceani* AT-1 (Accession number = OR905594) metabolites were acquired in SDF format from the PubChem database (https://pubchem.ncbi.nlm.nih.gov/;accessed on 01 January 2026). Energy minimization of ligands (metabolites) was executed using the OpenBabel tool of PyRx; protonation states were assigned at pH 7.4 and saved in PDBQT format [13]. For the comparison study, indole-acetic acid (IAA) and abscisic acid (ABA), as well-known plant growth hormones, were docked as reference compounds in the molecular docking study. For a robust comparison, the native ligand uridine-5-diphosphate (UDP) was also re-docked, and its Root Mean Square Deviation (RMSD) was calculated.

### Protein preparation and structure evaluation

In the current study, the structure of the maize regioselective silibinin glucosyltransferase enzyme, also known as UGT706F8, was selected as the target protein. The selected enzyme has been reported to transfer a glucose molecule to silibinin (a plant secondary metabolite having reactive oxygen species scavenging activity) to enhance its stability and solubility [14,23]. The three-dimensional protein structure (PDB ID: 7Q3S) was retrieved from the RCSB Protein Data Bank (https://www.rcsb.org). The target protein was prepared by removing extra ligands, water molecules, and heteroatoms, incorporating hydrogen atoms, protonation, energy minimization, and then saving in PDBQT format using the Macromolecule tool of PyRx [15]. The quality of the prepared protein structure was evaluated by PROCHECK Ramachandran plots.

### Ligand-protein docking protocol

The PyRx virtual screening tool was employed for molecular docking analysis to screen bacterial metabolites showing favorable molecular interactions with the target protein [15]. After ligand and protein preparation, the protein was loaded into PyRx, and a grid box was set with AutoGrid dimensions (Å) of (x = 60.6939, y = 63.9969, z = 66.9230) and with defined grid center coordinates (x = −6.2845, y = 0.1379, z = −19.0455), ensuring complete coverage of the binding site cavity. Grid box validation was executed by re-docking the native UDP ligand into the defined binding pocket. The docked pose closely reproduced the crystallographic orientation, yielding an RMSD of 0.9 Å, indicating that the selected grid box and docking protocol accurately reproduced the native binding pose and were suitable for subsequent screening of bacterial metabolites. The exhaustiveness value was kept at its default setting (i.e., 8). The target protein was kept rigid during the molecular docking procedure, while all rotatable bonds in the ligands were allowed flexible rotations. Three independent docking runs were carried out for each ligand to ensure reproducibility. Docked conformations with the lowest binding free energy (kcal/mol) were selected, and their binding conformations were visualized by PyMOL and BIOVIA Discovery Studio Visualizer (2021) software [16].

### Molecular dynamics simulation

Ligand-protein complex stability and conformational dynamics were investigated by molecular dynamics (MD) simulation analysis. On the basis of the lowest binding energies and favorable binding poses, two ligand-protein complexes were selected for MD simulation using Schrödinger’s Desmond software (version 2019−4) over 200 nanoseconds (ns) [17]. Two independent MD simulation runs were carried out for each ligand to ensure reproducibility. The energy minimization and optimization of ligand-protein complexes were achieved by Maestro or Schrödinger’s Protein Preparation Wizard. The system was prepared by applying the OPLS-2005 force field for molecular interactions [18,19]. Solvation was carried out by the TIP3P water model within an orthorhombic box via the System Builder, and the system was neutralized by adding counterions at a concentration of 0.15 M NaCl. MD simulations were performed under the NPT ensemble (i.e., 1 atm pressure and 310 K temperature) following an initial relaxation stage [17]. Principal Component (PC) analysis was also performed to visualize the dominant collective motions of protein-ligand complexes.

### Protein-protein interaction (PPI) network assessment

The STRING database (https://string-db.org; accessed on 01 March 2026) was employed to analyze protein-protein interactions to predict the potential biological context of the target protein [20]. The FASTA sequence of the target protein was retrieved from the protein database and submitted to the STRING server, and the species-specific interaction network was visualized by selecting *Zea mays* L. as the target organism. Furthermore, functional enrichment analysis was performed within the STRING database to predict associated biological pathways.

### Computational assessment of physicochemical properties, solubility and toxicity

The online web server SwissADME (https://www.swissadme.ch; accessed on 07 March 2026) was employed to evaluate the physicochemical parameters, including molecular weight, H-bond acceptors and donors, lipophilicity (LogP), and water solubility [21]. ProTox-3.0 was employed to predict the toxicity of selected metabolites [22].

## Results

### Protein structure evaluation

**Fig 2:**
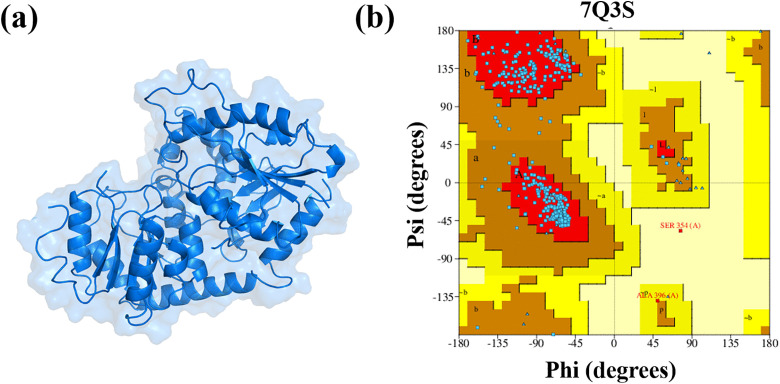
3D structure of the UGT706F8 protein, retrieved from the Protein Data Bank (a), and its Ramachandran plot generated by the PROCHECK web tool, displaying residue distribution (b).

The retrieved UGT706F8 protein consists of a single alpha-1 chain (A) (Fig 2a), having 488 amino acids, and is encoded on chromosome 9 of *Zea mays* L. cultivar. The PROCHECK Ramachandran plot suggested that most of the protein residues fall in a favorable region (89.2%), indicating favorable stereochemical quality (Fig 2b).

### Molecular docking findings

**Fig 3:**
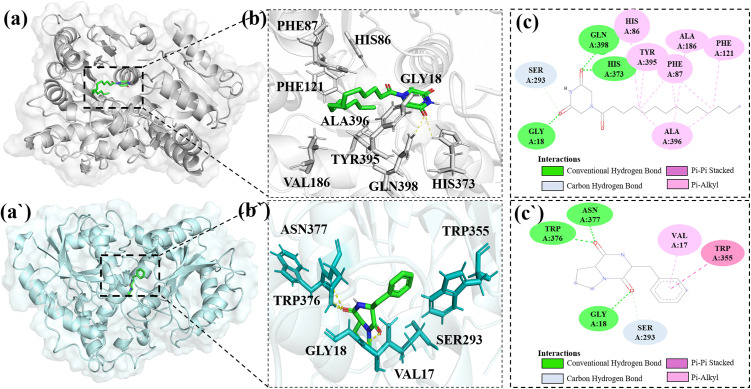
Molecular interaction of 4-tetradecanoyl-2,6-piperazinedione (a-c), and 3-Benzylhexahydropyrrolo (1,2-a)pyrazine-1,4-dione (a`-c`) with the active site of the target protein. Overall protein structure showing ligand (green stick) binding at the active site (a-a`), enlarged view of the binding pocket displaying key protein residues (b-b`), and 2D interactions of metabolites with the target protein (c-c`).

For virtual screening, 52 metabolites, synthesized by *Kocuria oceani* AT-1, were docked into the active site pocket of the maize UGT706F8 protein. The binding energies and protein residues involved in molecular interaction were presented in S1 Table in S1 File. All metabolites exhibited binding energies ranging from −4.3 to −8.5 kcal/mol. Based on the lowest binding energy and favorable binding pose, two compounds [4-tetradecanoyl-2,6-piperazinedione and 3-benzylhexahydropyrrolo(1,2-a)pyrazine-1,4-dione] were selected for further analyses and MD simulations (Table 1). Docking results showed that 4-tetradecanoyl-2,6-piperazinedione formed conventional hydrogen bonds with protein residues GLY18, HIS373, and GLN398, while SER293 was involved in carbon-hydrogen bonding with a docking score of −7.5 ± 0.33 kcal/mol. Moreover, amino acids HIS86, PHE87, PHE121, VAL186, TYR395, and ALA396 were involved in alkyl and pi-alkyl interactions as shown in Fig 3(a-a`). Similarly, 3-benzylhexahydropyrrolo(1,2-a)pyrazine-1,4-dione also formed hydrogen bonds with GLY18, TRP376, and ASN377 residues of the target protein, with a docking score of −8.5 ± 0.36 kcal/mol. Furthermore, SER293 participated in carbon-hydrogen bonding, while VAL17 and TRP355 were involved in pi-alkyl and pi-pi-stacked bonding, respectively, as shown in Fig 3(b-b`). The docking poses of control compounds were displayed in S1 Fig in S1 File, and chemical structures of selected metabolites were shown in S2 Fig in S1 File. Redocking of the native UDP ligand into the active site pocket of the UGT706F8 protein yielded a pose nearly identical to the co-crystallized ligand, with an RMSD of 0.9 Å (heavy atoms), reproducing the crystallographic binding orientation (S3, S4 Figs in S1 File).

**Table 1:** Computing binding energies (mean ± standard deviation from three independent molecular docking runs) and amino acids involved in molecular interactions between bacterial metabolites and UGT706F8 protein. Residue interactions are displayed for the representative lowest-energy docking pose.

Ligands	Compound CID	Binding energy (kcal/mol)	Interacted protein residues
Uridine-5-diphosphate (control)	6031	−9.5 ± 0.42	**Hydrogen bonds:** GLY18, SER293, HIS373, ASN377, SER378, GLN393, GLU397 **Pi-alkyl bonds:** VAL17 **Pi-pi stacked bonds:** TRP355 **Attractive charge:** ASP50
Indole-acetic acid (control)	802	−6.6 ± 0.34	**Hydrogen bonds:** ARG294, HIS373, GLN398 **Carbon hydrogen bonds:** GLY292, SER293 **Pi-alkyl bonds:** ALA396
Abscisic acid (control)	5280896	−5.8 ± 0.32	**Hydrogen bonds:** TRP355, GLN358
4-tetradecanoyl-2,6-piperazinedione	593506	−7.5 ± 0.33	**Hydrogen bonds:** GLY18, HIS373, GLN398 **Carbon hydrogen bonds:** SER293 **Alkyl and Pi-alkyl bonds:** HIS86, PHE87, PHE121, VAL186, TYR395, ALA396
3-benzylhexahydropyrrolo(1,2-a)pyrazine-1,4-dione	99895	−8.5 ± 0.36	**Hydrogen bonds:** GLY18, TRP376, ASN377 **Carbon hydrogen bonds:** SER293 **Pi-alkyl bonds:** VAL17 **Pi-pi stacked bonds:** TRP355

### Molecular dynamics simulation analysis

**Fig 4:**
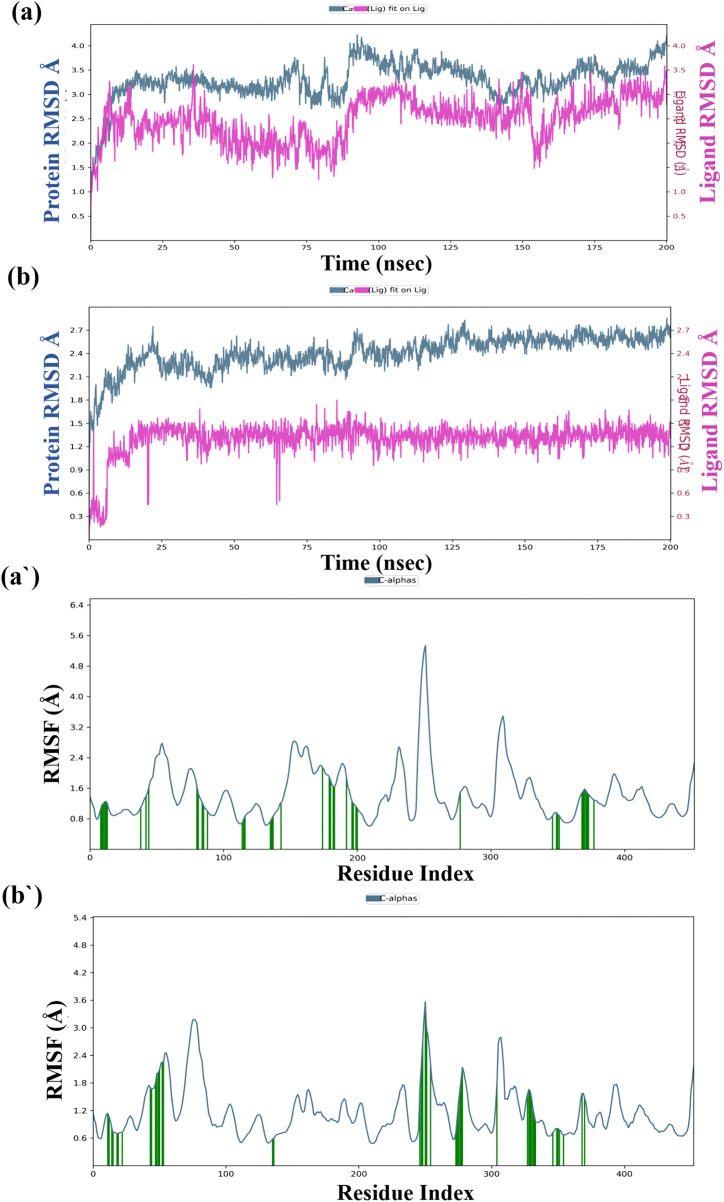
Root Mean Square Deviation (RMSD) and residue-wise Root Mean Square Fluctuation (RMSF) values of 4-tetradecanoyl-2,6-piperazinedione-protein complex (a-a`) and 3-benzylhexahydropyrrolo(1,2-a)pyrazine-1,4-dione-protein complex (b-b`) over 200 ns MD simulation.

Ligand-protein complex stability was evaluated by performing the MD simulation. The findings suggested that the ligand molecules remained stably bound within the protein active site pockets (Fig 4, S5 Fig in S1 File). During the 200 ns simulation period, no significant fluctuations in RMSD values were noted in both ligand-protein complexes. Specifically, in the 4-tetradecanoyl-2,6-piperazinedione-protein complex, the protein RMSD value showed an initial increase during the equilibrium phase and then stabilized, showing minor fluctuation around 3 to 3.8 Å after 75 ns. On the other hand, the ligand showed a minor fluctuation after 75 ns from 2 to 3 Å, indicating that the ligand remained within the active site pocket of the UGT706F8 protein (Fig 4a). Trajectory data of the 3-benzylhexahydropyrrolo(1,2-a)pyrazine-1,4-dione-protein complex showed that rapid fluctuation was observed in the protein backbone, followed by stabilization around 2 to 2.7 Å, while the ligand RMSD value remained very low around 1.2 to 3 Å, indicating limited movement within the protein active site pocket. These outcomes suggest that the ligand retained stable interactions with the protein residues over the course of the 200 ns simulation (Fig 4b). The outcomes of Root Mean Square Fluctuation (RMSF) of ligand-protein complexes displayed several fluctuation peaks over the 200 ns trajectory (Fig 4a`-b`). The low fluctuation observed for most residues suggests structural stability of the protein backbone. The key interacting residues fluctuated only slightly (approximately below 2 Å), indicating a stable ligand-protein interaction.

**Fig 5:**
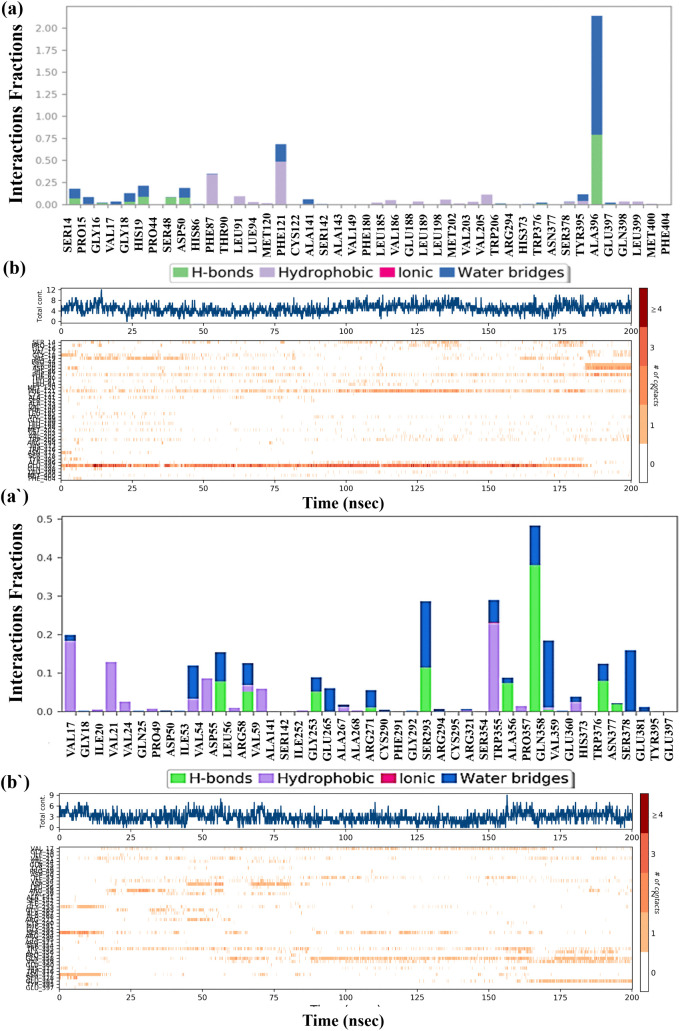
Protein-ligand interaction histograms and a timeline representation of 4-tetradecanoyl-2,6-piperazinedione-protein complex (a-a`) and 3-benzylhexahydropyrrolo(1,2-a)pyrazine-1,4-dione-protein complex (b-b`) showing hydrogen bonding, water bridge, and hydrophobic contacts.

The Protein-Ligand Interaction Fingerprints (PLIF) analysis of 4-tetradecanoyl-2,6-piperazinedione-protein complex demonstrated that the residues, including SER14, GLY16, GLY18, HIS19, SER48, ASP50, and GLU397 of the target protein, formed H-bonding with the ligand molecule, likely contributing to ligand-protein complex stability (Fig 5a). In the 3-benzylhexahydropyrrolo(1,2-a)pyrazine-1,4-dione-protein complex, ASP55, ARG58, GLY253, ARG271, SER293, ALA356, GLN358, ASN377, and SER378 were found to be key residues engaged in H-bond formation with the ligand molecule, contributing to stabilization of the ligand-protein complex (Fig 5, S6 Fig in S1 File).

**Fig 6:**
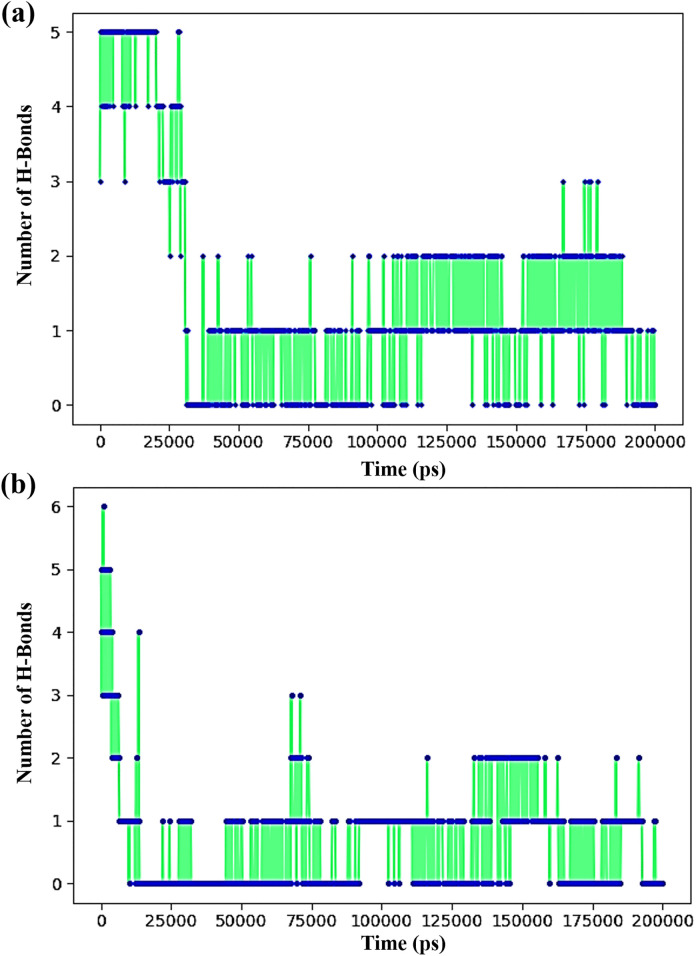
H-bond occupancy analysis of 4-tetradecanoyl-2,6-piperazinedione-protein complex (a) and 3-benzylhexahydropyrrolo(1,2-a)pyrazine-1,4-dione-protein complex (b) displaying hydrogen bonding over the course of a 200 ns simulation.

Hydrogen bond (H-bond) occupancy analysis displayed that both ligands formed favorable H-bonding over the course of a 200 ns simulation. The 4-tetradecanoyl-2,6-piperazinedione-protein complex showed peak occupancy reaching 5 H-bonds and maintained an average of 2.4 ± 0.6 H-bonds. Similarly, the 3-benzylhexahydropyrrolo(1,2-a)pyrazine-1,4-dione-protein complex maintained an average of 2.1 ± 0.5 H-bonds with peak occupancy reaching 6 H-bonds. These persistent H-bonds suggest stable molecular interactions with key protein residues (Fig 6, S7 Fig in S1 File).

**Fig 7:**
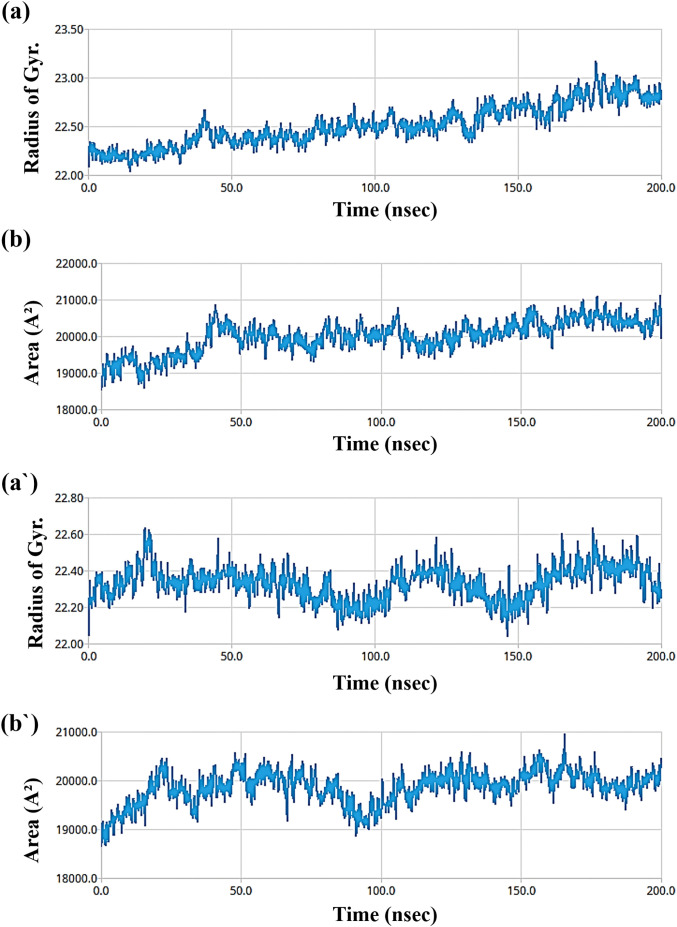
Radius of gyration (Rg) analysis and Solvent-Accessible Surface Area (SASA) profiles of 4-tetradecanoyl-2,6-piperazinedione-protein complex (a-a`) and 3-benzylhexahydropyrrolo(1,2-a)pyrazine-1,4-dione-protein complex (b-b`) showing minor conformational changes in UGT706F8 protein upon ligand binding over the course of a 200 ns simulation.

The Radius of gyration (Rg) of the 4-tetradecanoyl-2,6-piperazinedione-protein complex ranged between 22.0–23.5 Å over the course of a 200 ns simulation. The 3-benzylhexahydropyrrolo(1,2-a)pyrazine-1,4-dione-protein complex remained highly stable between 22.2–23.8 Å. The minimal fluctuations indicate that no major structural expansion was observed upon ligand binding. The Solvent-Accessible Surface Area (SASA) profiling of the 4-tetradecanoyl-2,6-piperazinedione-protein complex ranged between 19,000–22,000 Å² with minor fluctuations, while the 3-benzylhexahydropyrrolo(1,2-a)pyrazine-1,4-dione-protein complex ranged between 19,000–21,000 Å² with minor fluctuations, as shown in Fig 7 and S8 Fig in S1 File. The stable SASA profiles reflect that the UGT706F8 protein maintained its structural integrity without any major conformational changes upon ligand binding.

**Fig 8:**
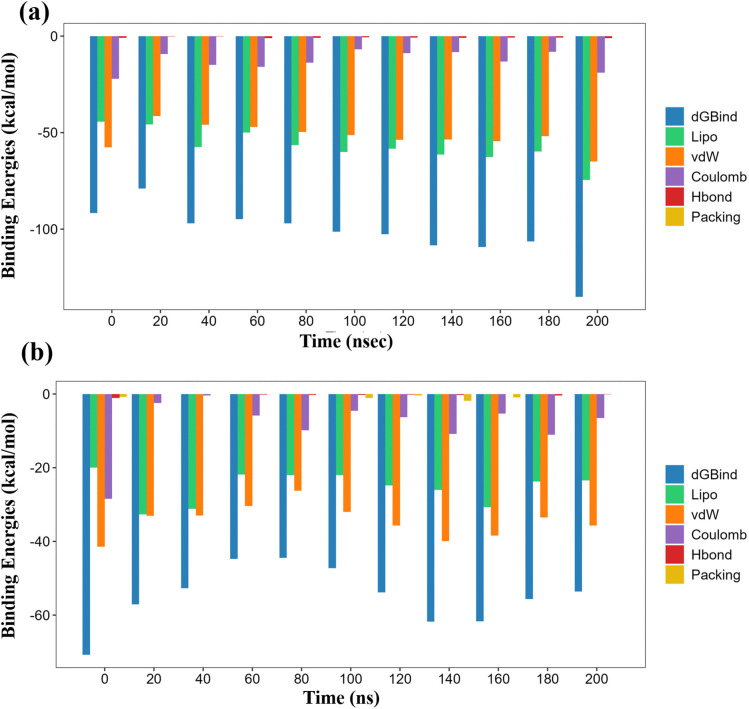
Molecular Mechanics Generalized Born Surface Area (MM-GBSA) analysis was conducted to predict binding free energies and energy components of 4-tetradecanoyl-2,6-piperazinedione-protein (a) and 3-benzylhexahydropyrrolo(1,2-a)pyrazine-1,4-dione-protein (b) complexes.

The ligand-protein complex stability was also supported by Molecular Mechanics Generalized Born Surface Area (MM-GBSA) analysis over the course of a 200 ns simulation. For the 4-tetradecanoyl-2,6-piperazinedione-protein complex, the average ΔG was found to be −102.05 kcal/mol with a standard deviation of 13.96, while the ΔG range was −91.64 to −135.13 kcal/mol. The mean ΔG for the 3-benzylhexahydropyrrolo(1,2-a)pyrazine-1,4-dione-protein complex was found to be −54.86 kcal/mol, with a range from −70.78 to −53.59 kcal/mol and a standard deviation of 7.93 (Fig 8, S9 Fig in S1 File).

**Fig 9:**
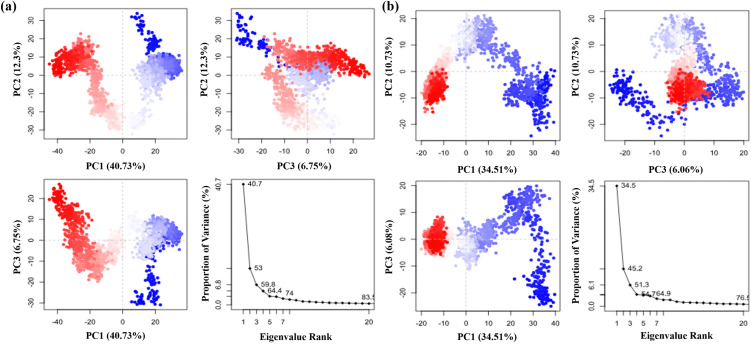
2D projection of Principal Component analysis of 4-tetradecanoyl-2,6-piperazinedione-protein complex (a) and 3-benzylhexahydropyrrolo(1,2-a)pyrazine-1,4-dione-protein complex (b). Each panel displays PC1-PC2, PC1-PC3, and PC2-PC3 projections, where the colors of the dots (red = final, blue = initial, and white = intermediate) indicate conformations by simulation time.

The Principal Component (PC) analysis of the 4-tetradecanoyl-2,6-piperazinedione-protein complex displayed that the first three principal components explained 60% of the variance (PC1 = 40.73%, PC2 = 12.3%, and PC3 = 6.75%), emphasizing the collective motion of the system (Fig 9a, S10 Fig in S1 File). Specifically, PCA outcomes of the 3-benzylhexahydropyrrolo(1,2-a)pyrazine-1,4-dione-protein complex revealed that the first three principal components explained 51% of the variance (PC1 = 34.51%, PC2 = 10.73%, and PC3 = 6.08%) (Fig 9b). The dominant PC1 fraction represented the conformational dynamics, where PC2 further highlighted the secondary motions, which may reflect local flexibility in surface regions. The fluctuation around PC2 and PC1 states was explained by the PC3 fraction. The separation of clusters was visualized in the fourth PC analysis plot, indicating a transition between metastable and stable states. These trajectory data suggest that protein-ligand interactions likely allow conformational adaptability of the protein.

**Fig 10:**
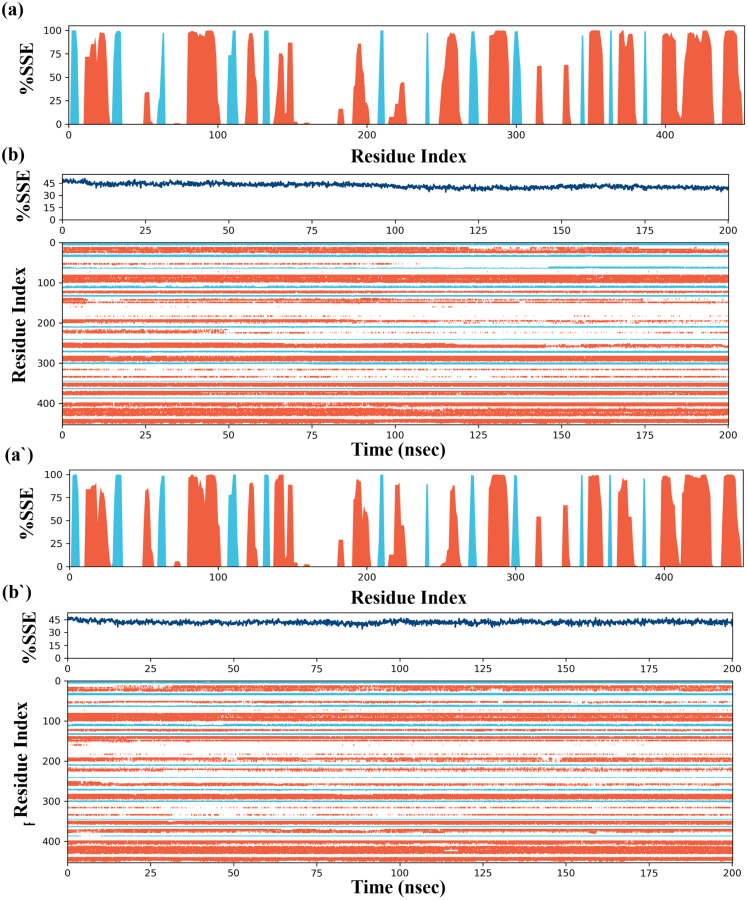
Dictionary of Secondary Structure of Proteins (DSSP) analysis of 4-tetradecanoyl-2,6-piperazinedione-protein (a) and 3-benzylhexahydropyrrolo(1,2-a)pyrazine-1,4-dione-protein (b) complexes, revealing the evolution of secondary structural elements over the simulation period.

Dictionary of Secondary Structure of Proteins (DSSP) analysis suggested stable secondary structure retention across both complexes, with α-helical content ranging from 42.3% to 45.8% and only minor fluctuations confined to loop regions, indicating the absence of global unfolding (Fig 10, S11 Fig in S1 File).

**Fig 11:**
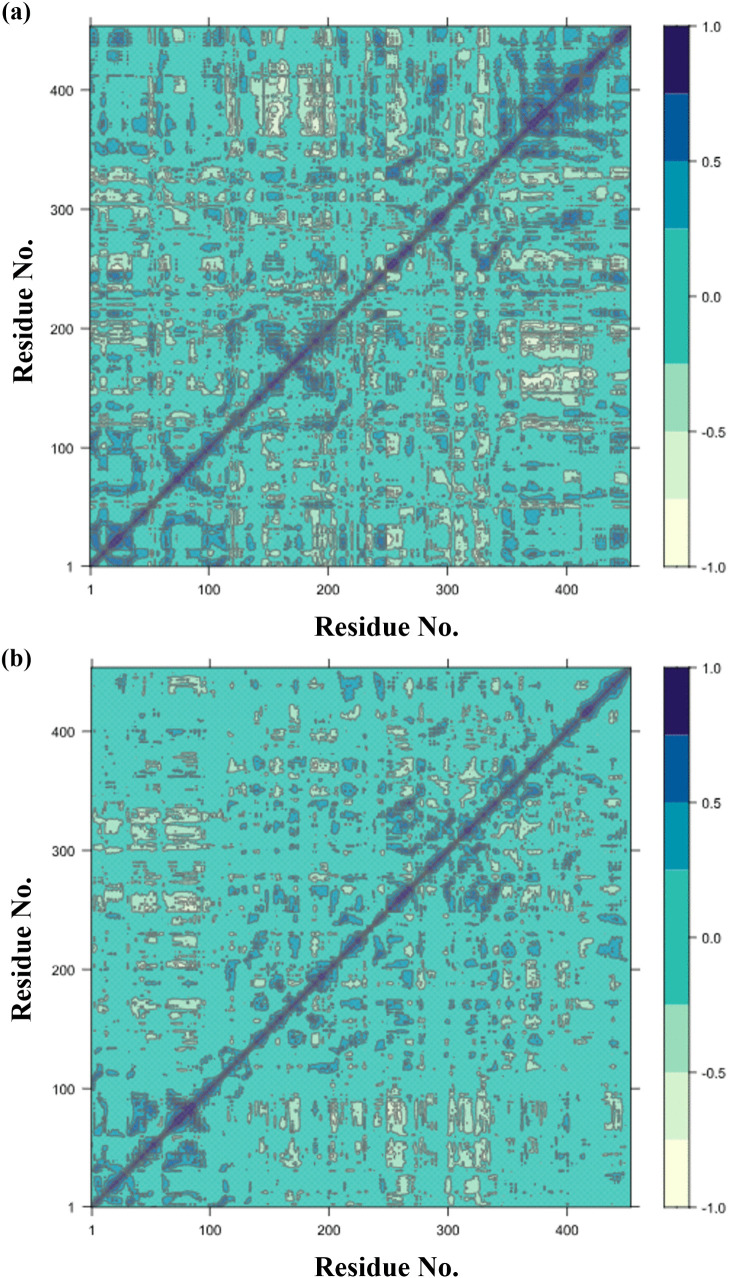
Dynamic Cross-Correlation Matrix (DCCM) analysis of 4-tetradecanoyl-2,6-piperazinedione-protein (a) and 3-benzylhexahydropyrrolo(1,2-a)pyrazine-1,4-dione-protein (b) complexes, displaying the correlated (positive) and anti-correlated (negative) residue motion during simulation.

Dynamic Cross-Correlation Matrix (DCCM) analysis revealed predominantly weak off-diagonal correlations (coefficients near zero), with few localized moderate couplings observed between the N- and C-terminal lobes (Fig 11, S12 Fig in S1 File).

### PPI network evaluation

**Fig 12:**
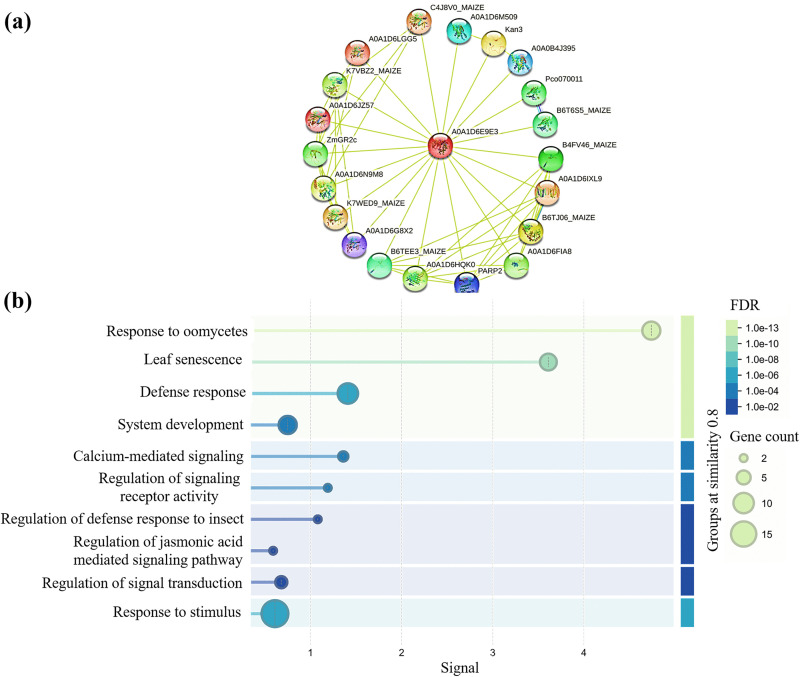
PPI network assessment of maize UGT706F8 protein (A0A1D6E9E3) illustrating potential interaction with key proteins in maize (a), and functional enrichment analysis of the target protein predicting important biological processes (b) by the STRING database.

To examine the potential biological context of the UGT706F8 protein, the protein-protein interaction (PPI) network, predicted by the STRING database, displayed a complex interaction pattern among proteins (Fig 12a and Table 2). Additionally, functional enrichment analysis predicted the potential involvement of the UGT706F8 protein in key cellular and metabolic pathways (Fig 12b).

**Table 2:** Protein-protein interaction of the UGT706F8 protein (A0A1D6E9E3) with other key regulatory proteins, indicating their predicted role in cellular and metabolic pathways.

S. No.	Protein ID	Confidence score	Protein description
1	A0A1D6IXL9	0.667	CMP-sialic acid transporter 4
2	B6TJ06_MAIZE	0.667	Glycosyltransferase family 28 C-terminal domain containing protein
3	A0A1D6HQK0	0.666	Legume lectins beta domain containing protein
4	B4FV46_MAIZE	0.663	Rapid alkalinization factor 1
5	B6TEE3_MAIZE	0.663	Rapid alkalinization factor 1
6	A0A1D6M509	0.660	Auxin transporter-like protein 1
7	A0A0B4J395	0.603	Auxin transporter-like protein 1
8	PARP2	0.559	Poly [ADP-ribose] polymerase 2
9	A0A1D6G8X2	0.556	Jasmonate-regulated gene 21
10	A0A1D6JZ57	0.556	Jasmonate-regulated gene 21
11	A0A1D6LG5	0.556	Jasmonate-regulated gene 21
12	C4J8V0_MAIZE	0.556	Jasmonate-regulated gene 21
13	K7WED9_MAIZE	0.556	Jasmonate-regulated gene 21
14	Kan3	0.518	Uncharacterized protein
15	A0A1D6N9M8	0.517	Methylesterase 3
16	K7VBZ2_MAIZE	0.517	Methylesterase 3
17	A0A1D6FIA8	0.499	Ubiquitin-like-specific protease ESD4
18	ZmGR2c	0.499	ZmGR2c protein
19	Pco070011	0.430	Galactose mutarotase-like superfamily protein
20	B6T6S5_MAIZE	0.430	Glucose-6-phosphate 1-epimerase

### Computational assessment of physicochemical properties, solubility and toxicity

The online web server SwissADME was employed for the assessment of physicochemical properties, solubility, and toxicity of selected metabolites. The predicted outcomes suggest that the selected metabolites have good physicochemical properties and solubility (Table 3). These metabolites were predicted to be non-carcinogenic, non-mutagenic, non-cytotoxic, and free from nutritional toxicity.

**Table 3:** Computational profiling of selected metabolites, representing their physicochemical properties, water solubility, lipophilicity, and toxicity.

Parameters		4-tetradecanoyl-2,6-piperazinedione	3-benzylhexahydropyrrolo-(1,2-a)pyrazine-1,4-dione
Physicochemical parameters	Molecular weight gm/mole	324.46 g/mol	244.29 g/mol
	Formula	C_18_H_32_N_2_O_3_	C_14_H_16_N_2_O_2_
	Fraction Csp3	0.83	0.43
	Molar Refractivity	100.65	74.76
	Aromatic heavy atoms	0	6
	Hydrogen bond donors	1	1
	Hydrogen bond acceptors	3	2
	Heavy atoms (count)	23	18
Lipophilicity	Log*P*_o/w_ (iLOGP)	3.42	2.03
	Log*P*_o/w_ (MLOGP)	2.33	1.18
	Log*P*_o/w_ (XLOGP3)	4.90	1.39
	Log*P*_o/w_ (SILICOS-IT)	4.26	1.61
	Log*P*_o/w_ (WLOGP)	2.41	−0.04
	Consensus Log *P*_o/w_	3.46	1.23
Water solubility	Log *S* (ESOL)	−4.08	−2.34
	Solubility	2.69e-02 mg/ml; 8.31e-05 mol/l	1.10e + 00 mg/ml; 4.52e-03 mol/l
	Class	Partially soluble	Soluble
	Log *S* (Ali)	−6.03	−2.03
	Solubility (mol/l)	3.01e-04 mg/ml; 9.29e-07 mol/l	2.27e + 00 mg/ml; 9.31e-03 mol/l
	Class	Slightly soluble	Soluble
	Log *S* (SILICOS-IT)	−5.33	−3.45
	Solubility	1.54e-03 mg/ml; 4.73e-06 mol/l	8.63e-02 mg/ml; 3.53e-04 mol/l
	Class	Partially soluble	Soluble
Toxicity	Mutagenicity	No	No
	Carcinogenicity	No	No
	Cytotoxicity	No	No
	Nutritional toxicity	No	No

## Discussion

In the current study, we employed virtual screening tools to predict the molecular interactions between *Kocuria oceani* AT-1 metabolites and maize protein (UGT706F8). The selected protein catalyzes the 7-OH glycosylation of silibinin (a plant-derived secondary metabolite belonging to the flavonolignan class) [14,23]. However, its selection as a target protein in the current study is based on its use in prior computational investigations [14] and its membership in the Family 1 UDP-glycosyltransferases (UGTs), which are well-documented in drought stress responses through phytohormone glycosylation and secondary metabolite modification [24–26]. Specifically, glycosylation of secondary metabolites such as silibinin and other flavonoids by UGTs enhances their stability, solubility, and antioxidant capacity [23,26]. Such modifications may contribute to reducing oxidative stress by regulating the activity and accumulation of plant protective secondary metabolites [14,23].

The outcomes of the molecular docking study revealed that all metabolites exhibited binding energy scores ranging from −4.3 to −8.5 kcal/mol (S1 Table in S1 File). Specifically, 4-tetradecanoyl-2,6-piperazinedione and 3-benzylhexahydropyrrolo(1,2-a)pyrazine-1,4-dione displayed favorable binding affinities for the target protein (Table 1, Fig 3) in comparison to plant growth hormones (S1 Fig in S1 File). The re-docked UDP compound exhibited a binding energy of −9.5 ± 0.42 kcal/mol and displayed several conventional hydrogen-bonding interactions, as shown in S3 Fig in S1 File. Several bacterial metabolites reproduced a similar interaction pattern, especially 4-tetradecanoyl-2,6-piperazinedione, establishing hydrogen-bonding with GLY18 and HIS373 residues of the protein, which were also observed in the UDP-protein complex. Comparably, 3-benzylhexahydropyrrolo(1,2-a) pyrazine-1,4-dione showed molecular interaction with VAL17, GLY18, SER293, TRP355, and ASN377 residues, which were also observed in the UDP-protein complex. Although the docking scores of the selected metabolites were comparable to the control compounds, the favorable predicted binding orientations observed within the UGT706F8 active site suggest that *Kocuria oceani* AT-1 metabolites may establish stable molecular interactions with maize protein.

To support molecular docking outcomes, we analyzed the stability of metabolite-protein complexes by MD simulation over 200 ns [19,27,28]. The results revealed that both ligands remained stable during the 200 ns simulation. The 4-tetradecanoyl-2,6-piperazinedione-protein complex achieved stability after ~75 ns, with the protein backbone showing fluctuations in RMSD value between 3.0–3.8 Å. However, the ligand RMSD value remained stable between 2.0–3.0 Å, suggesting ligand-protein complex stability (Fig 4a, S5 Fig in S1 File) [29–31]. Comparably, the 3-benzylhexahydropyrrolo(1,2-a)pyrazine-1,4-dione-protein complex reached stability around 2–2.7 Å with a very low ligand RMSD fluctuation around 1.2−3 Å, indicating very limited movement inside the protein pocket (Fig 4b). Similarly, RMSF values of key interacting residues of both complexes showed lower fluctuations throughout the simulation period, suggesting good structural integrity and stability of the protein (Fig 4b-b`) [32,33]. The persistent molecular interactions between the ligand and UGT706F8 protein were supported by PLIF analysis and H-bond occupancy analysis (Fig 5, S6 Fig in S1 File) [34,35]. The 4-tetradecanoyl-2,6-piperazinedione-protein complex maintained an average of 2.4 ± 0.6 H-bonds, while an average of 2.1 ± 0.5 H-bonds was observed in the 3-benzylhexahydropyrrolo(1,2-a)pyrazine-1,4-dione-protein complex (Fig 6, S7 Fig in S1 File). Minor fluctuations were observed in the Rg values ranged between 22.0–23.5 Å, and 22.2–23.8 Å, respectively, and SASA profiles ranged between 19,000–22,000 Å^2^, and 19,000–21,000 Å^2^, respectively, suggesting that the protein did not undergo major conformational changes upon ligand binding and remained stable throughout the simulation (Fig 7, S8 Fig in S1 File). Additionally, MM-GBSA calculations revealed that both metabolites exhibited favorable binding energies (ΔG_bind_) (−102.05 ± 13.96 and −54.86 ± 7.93 kcal/mol, respectively), suggesting favorable binding energetics (Fig 8, S9 Fig in S1 File). The predicted high van der Waals contributions (−51.95 ± 6.4 and −34.48 ± 4.3, respectively) suggest favorable molecular interactions. The PC analysis projection further suggests that the *Kocuria oceani* AT-1 metabolite may stabilize the UGT706F8 protein in a specific conformational ensemble (Fig 9, S10 Fig in S1 File). Stable α-helical content (42–46%) and minimal loop fluctuations suggest that the UGT706F8 protein retains its native fold upon ligand binding without global unfolding (Fig 10, S11 Fig in S1 File). Weak inter-residue correlations indicate a rigid scaffold, while noticeable rigidification upon ligand binding suggests reduced conformational entropy that correlates with favorable MM-GBSA binding energy (Fig 11, S12 Fig in S1 File).

These outcomes highlight the potential of these metabolites as computationally predicted candidates that could be explored as structurally interesting leads for future experimental studies [36,37]. In previous studies, researchers used bacterial metabolites such as 2,3-butanediol [38], cyclic peptide [39,40], and 3-hydroxy-2-butanone [41] to enhance plant resilience to environmental stressors. The application of 3-benzylhexahydropyrrolo(1,2-a)pyrazine-1,4-dione [a cyclic peptide known as cyclo(Phe-Pro)] as a potential drought-mitigating lead compound is supported by the outcomes of a recent study revealing that the related cyclic peptide [cyclo(Leu-Pro)] potentially boosted maize resilience to drought stress by modulating the antioxidant defense system [40], suggesting that cyclic peptides could be promising candidates for strengthening crop resilience. Exploring PGPB-derived metabolites to improve abiotic stress tolerance in cereal crops could be a promising strategy for sustaining agricultural productivity and combating food scarcity under changing climatic conditions [42–46].

Additionally, PPI network analysis suggested that the UGT706F8 protein may interact with several key proteins as predicted by the STRING database (Fig 12). The predicted interactions showed that the RALF peptide (stimulates stomata closing through the abscisic acid pathway), jasmonate-regulated protein (modulates the antioxidant defense system), auxin transporter (enhances water uptake by modulating root architecture), and PAPR2 (preserves DNA integrity under oxidative stress) (Table 2) proteins are linked with the UGT706F8 protein. However, the biological significance of bacterial metabolites binding to the UGT706F8 protein and their influence on associated proteins remain speculative and require experimental validation. Furthermore, these metabolites were predicted to have good physicochemical properties and solubility as well as predicted to be non-carcinogenic, non-cytotoxic, and non-mutagenic (Table 3).

The predicted interactions between bacterial metabolites and the UGT706F8 protein are based solely on computational analyses, which is a limitation of the present study. Although UGT706F8 protein has been associated with flavonoid glycosylation, *in vivo* experimental evidence demonstrating that modulation of UGT706F8 protein boosts maize resilience to drought stress remains unavailable. Therefore, the observed binding affinities and dynamics simulation findings should be interpreted as indicators of potential molecular interactions rather than proof of a causal effect on drought resilience. Expanding these analyses to a wider range of drought-responsive proteins in future studies may provide deeper insights into the potential role of microbial metabolites in maize stress adaptation. Future experimental validation should encompass *in vitro* enzyme activity assays, qRT-PCR-based gene expression analysis, LC-MS/MS metabolomics profiling, and greenhouse drought experiments to assess plant responses, including physiological, biochemical, and photosynthetic efficiency. Nevertheless, our study emphasizes the significance of computational approaches in identifying, designing, and selecting effective candidate compounds before any *in vivo* trial, thereby contributing to the development of eco-friendly agriculture approaches.

## Conclusion

Challenges associated with limited irrigation in drought-prone regions and low rainfall continue to hinder traditional farming practices. Among these hurdles, soil water deficit amplifies the vulnerability of cereal crops. To address these challenges, our computational study provides molecular-level insights into the potential interactions of *Kocuria oceani*-derived metabolites with maize glycosyltransferase UGT706F8, an enzyme associated with flavonoid glycosylation. Among *Kocuria oceani* AT-1 metabolites, 4-tetradecanoyl-2,6-piperazinedione and 3-benzylhexahydropyrrolo(1,2-a)pyrazine-1,4-dione displayed favorable binding energies (−7.5 ± 0.33 and −8.5 ± 0.36 kcal/mol, respectively), and maintained stable interactions throughout the molecular dynamics simulation, identifying them as computationally predicted candidates for future investigation. However, further *in vivo* validation is required to elucidate the mechanistic pathways of these metabolites, to determine their optimal concentration for in-field application, and to elucidate their synergistic effects with other natural bioactive compounds. Collectively, these findings provide a computational framework for predicting microbial metabolites for future experimental evaluation as potential candidates for climate-resilient agricultural strategies.

## Supporting information

S1 File S1 Table. Binding energies are expressed as mean ± SD from three independent docking runs, whereas the interacting protein residues are derived from the representative lowest-energy docking pose. **S1 Fig.** 2D and 3D molecular interaction of indole acetic acid (a-a`), and abscisic acid (b-b`) with target protein residues. **S2 Fig.** Chemical structures of 4-tetradecanoyl-2,6-piperazinedione, (a) and 3-benzylhexahydropyrrolo(1,2-a)pyrazine-1,4-dione (b) as selected ligand molecules. **S3 Fig.** 2D and 3D molecular interaction of uridine-5-diphosphate with target protein residues after re-docking. **S4 Fig.** Superimposition of the re-docked UDP (Red) with the co-crystallized UDP (Blue) within the binding pocket of UGT706F8 (PDB: 7Q3S). The RMSD of 0.9 Å (heavy atoms) between the two poses validates the reproducibility of the docking protocol. **S5 Fig.** RMSD and residues-wise RMSF values of 4-tetradecanoyl-2,6-piperazinedione-protein complex (a-a`) and 3-benzylhexahydropyrrolo(1,2-a)pyrazine-1,4-dione-protein complex (b-b`) over 200 ns MD simulation. **S6 Fig.** Protein-ligand interaction histograms and a timeline representation of 4-tetradecanoyl-2,6-piperazinedione-protein complex (a-a`) and 3-benzylhexahydropyrrolo(1,2-a)pyrazine-1,4-dione-protein complex (b-b`) showing hydrogen bonding, water bridge, and hydrophobic contacts. **S7 Fig.** H-bond occupancy analysis of 4-tetradecanoyl-2,6-piperazinedione-protein complex (a) and 3-benzylhexahydropyrrolo(1,2-a)pyrazine-1,4-dione-protein complex (b) displaying hydrogen bonding over the course of a 200 ns simulation. **S8 Fig.** Rg analysis and SASA profiles of 4-tetradecanoyl-2,6-piperazinedione-protein complex (a-a`) and 3-benzylhexahydropyrrolo(1,2-a)pyrazine-1,4-dione-protein complex (b-b`) showing minor conformational changes in UGT706F8 protein upon ligand binding over the course of a 200 ns simulation. **S9 Fig.** MM-GBSA analysis was conducted to predict binding free energies and energy components of 4-tetradecanoyl-2,6-piperazinedione-protein (a) and 3-benzylhexahydropyrrolo(1,2-a)pyrazine-1,4-dione-protein (b) complexes. **S10 Fig.** 2D projection of Principal Component analysis of 4-tetradecanoyl-2,6-piperazinedione-protein complex (a) and 3-benzylhexahydropyrrolo(1,2-a)pyrazine-1,4-dione-protein complex (b). Each panel displays PC1-PC2, PC1-PC3, and PC2-PC3 projections, where the colors of the dots (red = final, blue = initial, and white = intermediate) indicate conformations by simulation time. **S11 Fig.** Dictionary of Secondary Structure of Proteins (DSSP) analysis of 4-tetradecanoyl-2,6-piperazinedione-protein (a) and 3-benzylhexahydropyrrolo(1,2-a)pyrazine-1,4-dione-protein (b) complexes, revealing the evolution of secondary structural elements over the simulation period. **S12 Fig.** Dynamic Cross-Correlation Matrix (DCCM) analysis of 4-tetradecanoyl-2,6-piperazinedione-protein (a) and 3-benzylhexahydropyrrolo(1,2-a)pyrazine-1,4-dione-protein (b) complexes, displaying the correlated (positive) and anti-correlated (negative) residue motion during simulation.
(DOCX)
