# Supplementary material for: Virtual screening of Kocuria oceani AT-1 metabolites as potential maize growth regulators under drought conditions using molecular docking and dynamics simulation
Source: PLoS One. 2026 Jul 24;21(7):e0354805. doi: 10.1371/journal.pone.0354805 (PMC13399325; doi:10.1371/journal.pone.0354805)
Supplement: S1 File — S1 Fig. 2D and 3D molecular interaction of indole acetic acid (a-a`), and abscisic acid (b-b`) with target protein residues. S2 Fig. Chemical structures of 4-tetradecanoyl-2,6-piperazinedione, (a) and 3-benzylhexahydropyrrolo(1,2-a)pyrazine-1,4-dione (b) as selected ligand molecules. S3 Fig. 2D and 3D molecular interaction of uridine-5-diphosphate with target protein residues after re-docking. S4 Fig. Superimposition of the re-docked UDP (Red) with the co-crystallized UDP (Blue) within the binding pocket of UGT706F8 (PDB: 7Q3S). The RMSD of 0.9 Å (heavy atoms) between the two poses validates the reproducibility of the docking protocol. S5 Fig. RMSD and residues-wise RMSF values of 4-tetradecanoyl-2,6-piperazinedione-protein complex (a-a`) and 3-benzylhexahydropyrrolo(1,2-a)pyrazine-1,4-dione-protein complex (b-b`) over 200 ns MD simulation. S6 Fig. Protein-ligand interaction histograms and a timeline representation of 4-tetradecanoyl-2,6-piperazinedione-protein complex (a-a`) and 3-benzylhexahydropyrrolo(1,2-a)pyrazine-1,4-dione-protein complex (b-b`) showing hydrogen bonding, water bridge, and hydrophobic contacts. S7 Fig. H-bond occupancy analysis of 4-tetradecanoyl-2,6-piperazinedione-protein complex (a) and 3-benzylhexahydropyrrolo(1,2-a)pyrazine-1,4-dione-protein complex (b) displaying hydrogen bonding over the course of a 200 ns simulation. S8 Fig. Rg analysis and SASA profiles of 4-tetradecanoyl-2,6-piperazinedione-protein complex (a-a`) and 3-benzylhexahydropyrrolo(1,2-a)pyrazine-1,4-dione-protein complex (b-b`) showing minor conformational changes in UGT706F8 protein upon ligand binding over the course of a 200 ns simulation. S9 Fig. MM-GBSA analysis was conducted to predict binding free energies and energy components of 4-tetradecanoyl-2,6-piperazinedione-protein (a) and 3-benzylhexahydropyrrolo(1,2-a)pyrazine-1,4-dione-protein (b) complexes. S10 Fig. 2D projection of Principal Component analysis of 4-tetradecanoyl-2,6-piperazinedione-protein complex (a) a [file pone.0354805.s001.docx]

**Supplementary data**

**S1 Table.** Binding energies are expressed as mean ± SD from three independent docking runs, whereas the interacting protein residues are derived from the representative lowest-energy docking pose.

| **Sr. no** | **Docked ligands** | **Compound CID** | **Binding energies (kcal/mol)** | **Protein residues involved in molecular interaction** |
| --- | --- | --- | --- | --- |
| 1 | 3-Benzylhexahydropyrrolo(1,2-a)pyrazine-1,4-dione | 99895 | -8.5±0.36 | **Hydrogen bonds:** GLY18, TRP376, ASN377  **Carbon hydrogen bonds:** SER293  **Pi-alkyl bonds:** VAL17  **Pi-pi stacked bonds:** TRP355 |
| 2 | 4-Tetradecanoyl-2,6-piperazinedione | 593506 | -7.5±0.33 | **Hydrogen bonds:** GLY18, HIS373, GLN398  **Carbon hydrogen bonds:** SER293  **Alkyl and** **Pi-alkyl bonds:**  HIS86, PHE87, PHE121, VAL186, TYR395, ALA396 |
| 3 | 3-Isobutylhexahydropyrrolo(1,2-a)pyrazine-1,4-dione | 102892 | -7.4±0.32 | **Conventional hydrogen bonds:** GLY18, TRP376, ASN377  **Alkyl and Pi-alkyl bonds:** VAL17, TRP355, HIS373, |
| 4 | Naphthalene, 1-methyl- | 7002 | -6.9±0.05 | **Alkyl and Pi-alkyl bonds:** PRO13, PRO15, ILE20, ILE41, PRO44, VAL54 |
| 5 | Naphthalene, 1,5-dimethyl- | 11306 | -6.9±1.08 | **Alkyl and Pi-alkyl bonds:** VAL17  **Pi-sigma bonds:** TRP355  **Pi anion bonds:** GLU381 |
| 6 | Naphthalene, 1,8-dimethyl- | 11287 | -6.7±0.05 | **Alkyl bonds:** VAL186, ALA396  **Pi anion bonds:** GLU397 |
| 7 | 2,4-di-t-Butylphenol | 7311 | -6.5±0.30 | **Alkyl and Pi-alkyl bonds:** VAL21, VAL24, VAL54, VAL59 |
| 8 | 3,6-Diisobutyl-2,5-piperazinedione | 137006 | -6.3±0.80 | **Conventional hydrogen bonds:** LYS28  **Alkyl bonds:** VAL21, VAL24, VAL54, VAL59, VAL359 |
| 9 | 3,6-Diisopropylpiperazin-2,5-dione | 519728 | -6.2±0.63 | **Conventional hydrogen bonds:** VAL17, GLY18, ASN377, SER378  **Carbon hydrogen bonds:** GLY375 |
| 10 | Palmitic acid, beta-monoglyceride | 123409 | -6.1±0.2 | **Conventional hydrogen bonds:** TRP376, ASN377  **Carbon hydrogen bonds:** HIS373  **Alkyl and Pi-alkyl bonds:** VAL17, VAL21, PRO357, VAL359 |
| 11 | Pyyrolidino(1,2-a)piperazine-3,6-dione | 193540 | -6.1±1.08 | **Conventional hydrogen bonds:** HIS19, TRP376, GLU397  **Carbon hydrogen bonds:** GLY375  **Pi-alkyl bonds:** HIS373 |
| 12 | Undecane, 3,6-dimethyl- | 86539 | -6.0±0.69 | **Alkyl and Pi-alkyl bonds:** HIS19, HIS85, PHE87, PHE121, VAL186, TRP206, ALA396 |
| 13 | Benzenepropanoic acid | 107 | -5.9±0.24 | **Conventional hydrogen bonds:** GLU43, ARG52  **Alkyl bonds:** PRO13, PRO15, ILE20, ILE41, PRO44 |
| 14 | Tridecane,2-methyl- | 15269 | -5.8±0.23 | **Alkyl and Pi-alkyl bonds:** HIS19, HIS85, PHE87, PHE121, VAL186, LEU198, MET202, TRP206, TYR395, ALA396 |
| 15 | Oleic acid | 445639 | -5.8±0.56 | **Conventional hydrogen bonds:** ASN377  **Alkyl and Pi-alkyl bonds:** PHE87, PHE121, VAL186, MET202, TRP206, HIS373, TYR395, ALA396 |
| 16 | Pentadecane,2,6,10,14-tetramethyl- | 15979 | -5.8±0.58 | **Alkyl and Pi-alkyl bonds:** VAL17, VAL21, VAL24, VAL54, VAL59, TRP355, VAL359 |
| 17 | Benzonitrile,2-amino-4,5-diethoxy- | 1381991 | -5.7±0.65 | **Conventional hydrogen bonds:** ARG294, TYR395  **Alkyl and Pi-alkyl bonds:** HIS19, MET120, PHE121, HIS373, ALA396 |
| 18 | 4-(1-Pyyrolidinyl)-1,5-dihydro-2H-pyyrol-2-one | 592358 | -5.7±0.0 | **Conventional hydrogen bonds:** SER293, ARG294, TYR395  **Alkyl bonds:** ALA396 |
| 19 | Eicosanoic acid | 10467 | -5.6±0.41 | **Conventional hydrogen bonds:** ASN377  **Alkyl and Pi-alkyl bonds:** HIS19, HIS86, PHE87, PHE121, VAL186, MET202, TRP206, TYR395, ALA396 |
| 20 | N,N-Dimethyldecanamide | 26690 | -5.5±0.25 | **Alkyl and Pi-alkyl bonds:** HIS85, PHE87, PHE121, VAL186, MET202, TRP206, ALA396 |
| 21 | Hexadecenoic acid, 2-hydroxy-1-(hydroxymethyl)ethyl ester | 53745895 | -5.5±0.23 | **Conventional hydrogen bonds:** GLN358, HIS373  **Alkyl and Pi-alkyl bonds:** VAL17, VAL54, VAL359  **Pi-sigma bonds:** TRP355 |
| 22 | Dodecane, 2,6,10-trimethyl- | 19773 | -5.4±0.43 | **Alkyl and Pi-alkyl bonds:** HIS19, PHE87, PHE121, VAL186, TYR395, ALA396 |
| 23 | Dodecane,2,7,10-trimethyl- | 93447 | -5.4±0.56 | **Alkyl and Pi-alkyl bonds:** PHE87, PHE121, ALA141, VAL186, MET202, TRP206, TYR395, ALA396  **Pi-sigma bonds:**  HIS19 |
| 24 | 1-Heneicosyl formate | 545651 | -5.3±0.60 | **Conventional hydrogen bonds:** SER293, GLN358  **Carbon hydrogen bonds:** GLY292  **Alkyl and Pi-alkyl bonds:** VAL17, VAL21, VAL24, VAL54, VAL59, TRP355, VAL359 |
| 25 | Heptadecane | 12398 | -5.3±0.40 | **Alkyl and Pi-alkyl bonds:** HIS19, PHE87, PHE121, VAL186, MET202, TYR395, ALA396 |
| 26 | Pentadecanoic acid | 13849 | -5.3±0.68 | **Conventional hydrogen bonds:** TRP376, ASN377, GLU397  **Alkyl and Pi-alkyl bonds:** HIS19, PHE121, MET202, TYR395, ALA396  **Pi-sigma bonds:** PHE87 |
| 27 | Tetradecanoic acid | 11005 | -5.3±0.56 | **Conventional hydrogen bonds:** TRP376, ASN377, GLU397  **Carbon hydrogen bonds:** GLY375  **Alkyl and Pi-alkyl bonds:** HIS19, PHE87, PHE121, VAL186, MET202, TRP206, ALA396 |
| 28 | Dodecanoic acid | 3893 | -5.3±0.46 | **Alkyl bonds:** PRO13, PRO15, ILE20, ILE41, PRO44, VAL54, LEU56 |
| 29 | 2-Heptanone, 3-methyl- | 92927 | -5.2±0.21 | **Conventional hydrogen bonds:** ARG52  **Carbon hydrogen bonds:** PRO44 |
| 30 | 1,4-Cyclohexanedione | 12511 | -5.2±0.0 | **Conventional hydrogen Bonds:** ARG52  **Carbon hydrogen bonds:** PRO44 |
| 31 | 1-Heptadecanol | 15076 | -5.2±0.19 | **Alkyl and Pi-alkyl bonds:** VAL17, VAL21, VAL24, VAL54, VAL59, TRP355 |
| 32 | 3-Pyrrolidin-2-yl-propionic acid | 550965 | -5.2±0.05 | **Conventional hydrogen bonds:** SER142, ASN377, GLU397  **Alkyl and Pi-alkyl bonds:** HIS19, ALA396 |
| 33 | Octadecanoic acid | 5281 | -5.1±0.35 | **Conventional hydrogen bonds:** GLU381  **Carbon hydrogen bonds:** GLY18  **Alkyl and Pi-alkyl bonds:** VAL17, VAL21, VAL24, VAL54, VAL59, TRP355 |
| 34 | 1-Decanol, 2-hexyl- | 95337 | -5.1±0.03 | **Conventional hydrogen bonds:** GLN358  **Alkyl and Pi-alkyl bonds:** VAL17, VAL21, VAL54, CYS290  **Pi-sigma bonds:** TRP355 |
| 35 | Tridecanoic acid | 12530 | -5.1±0.23 | **Conventional hydrogen bonds:** HIS19  **Alkyl and Pi-alkyl bonds:** PHE87, PHE121, VAL186, TRP206, TYR395, ALA396 |
| 36 | Caprolactam | 7768 | -5.0±0.21 | **Conventional hydrogen bonds:** ASP50  **Carbon hydrogen bonds:** PRO15  **Alkyl bonds:** ILE20, ILE41, VAL54, LEU56 |
| 37 | Pentadecane | 12391 | -4.9±0.17 | **Alkyl and Pi-alkyl bonds:** VAL17, VAL21, VAL24, VAL54, VAL59, TRP355, VAL359 |
| 38 | 10-Methyl-octadec-1-ene | 545557 | -4.9±0.61 | **Alkyl and Pi-alkyl bonds:** VAL17, VAL21, VAL24, VAL54, VAL59, TRP355, PRO357, VAL359 |
| 39 | Undecane, 2-methyl- | 23459 | -4.9±0.05 | **Alkyl and Pi-alkyl bonds:** HIS19, PHE87, PHE121, VAL186, MET202, TRP206, ALA396 |
| 40 | Octadecane | 11635 | -4.8±0.32 | **Alkyl bonds:** VAL17, VAL21, VAL24, VAL54, VAL59, VAL359 |
| 41 | Decane, 1,1′-oxybis- | 17152 | -4.8±0.20 | **Alkyl and Pi-alkyl bonds:** VAL17, VAL21, VAL24, VAL54, VAL59, TRP355, VAL359, HIS373 |
| 42 | Hexadecane | 11006 | -4.8±0.45 | **Alkyl and Pi-alkyl bonds:** VAL17, ILE20, VAL21, VAL24, VAL54, VAL59, TRP355 |
| 43 | Heneicosane | 12403 | -4.7±0.36 | **Alkyl and Pi-alkyl bonds:** VAL17, VAL21, VAL24, VAL54, VAL59, TRP355, HIS373 |
| 44 | Nonadecane | 12401 | -4.7±0.80 | **Alkyl and Pi-alkyl bonds:** VAL21, VAL24, VAL54, VAL59, TRP355, VAL359 |
| 45 | Undecane | 14257 | -4.6±0.11 | **Alkyl and Pi-alkyl bonds:** HIS19, PHE87, PHE121, VAL186, MET202, TYR395, ALA396 |
| 46 | Docosane | 12405 | -4.6±0.05 | **Alkyl bonds:** VAL17, VAL21, VAL24, VAL54, VAL59, VAL359 |
| 47 | 2-Piperidone | 12665 | -4.6±0.05 | **Conventional hydrogen bonds:** ARG52, ILE53, VAL54  **Alkyl bonds:** ILE20, ILE41 |
| 48 | 3-Penten-2-one, 4-methyl- | 8858 | -4.6±0.05 | **Conventional hydrogen bonds:** ALA42  **Carbon hydrogen bonds:** ILE41  **Alkyl bonds:** ILE20, VAL54, LEU56 |
| 49 | Dodecane | 8182 | -4.5±0.43 | **Alkyl and Pi-alkyl bonds:** HIS19, PHE87, PHE121, VAL186, MET202, ALA396 |
| 50 | Tridecane | 12388 | -4.5±0.23 | **Alkyl and Pi-alkyl bonds:** PHE87, PHE121, VAL186, TRP206, TYR395, ALA396 |
| 51 | Dodecane,4,6-dimethyl- | 545627 | -4.5±0.25 | **Alkyl and Pi-alkyl bonds:** HIS85, HIS86, PHE87, PHE121, VAL186, LEU198, MET202, TRP206, ALA396  **Pi-sigma bonds:** TYR395 |
| 52 | Tetradecane | 12389 | -4.3±0.05 | **Alkyl and Pi-alkyl bonds:** HIS19, PHE87, PHE121, VAL186, TYR395, ALA396 |

| **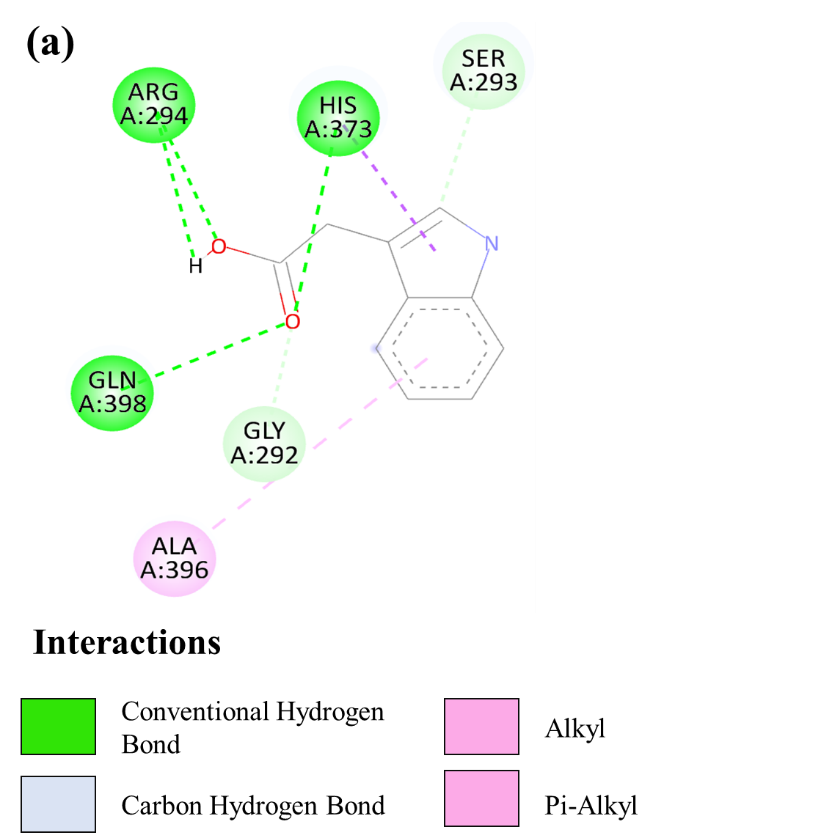** | **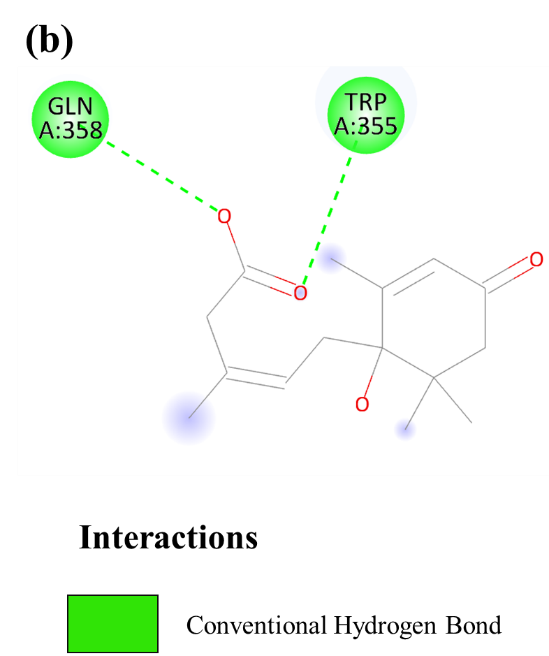** |
| --- | --- |
| **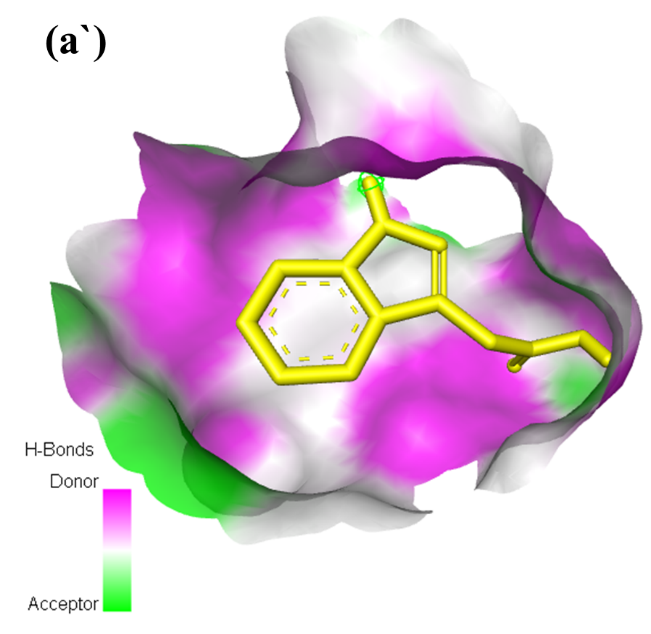** | **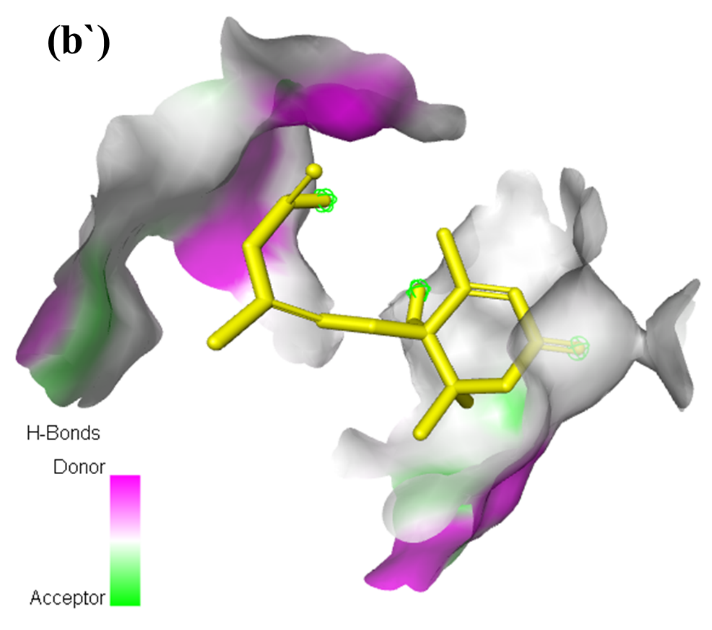** |

**S1 Fig.** 2D and 3D molecular interaction of indole acetic acid (a-a`), and abscisic acid (b-b`) with target protein residues.


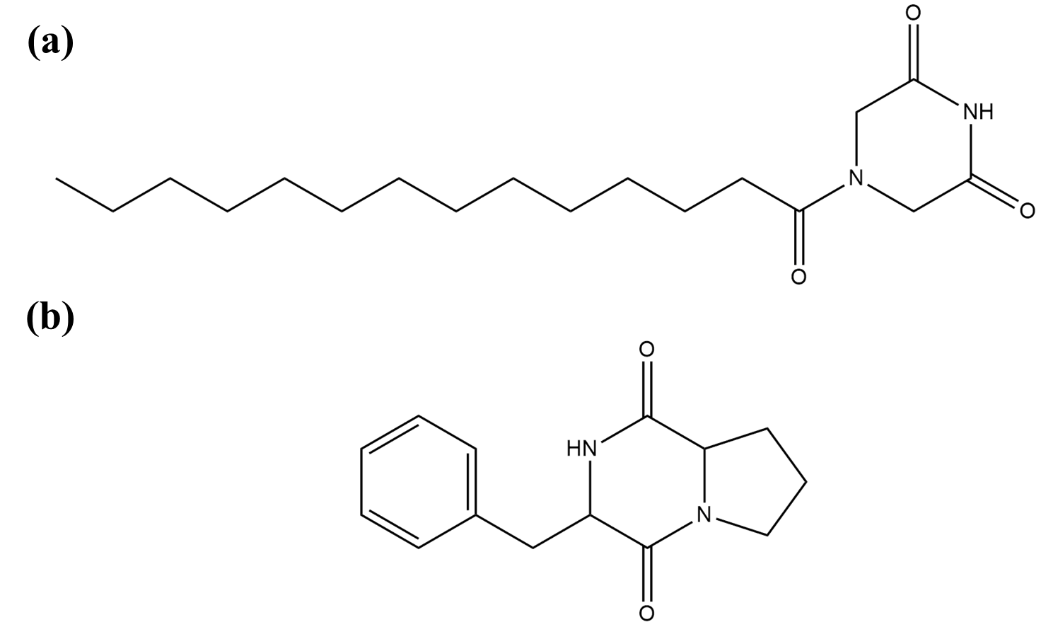


**S2 Fig.** Chemical structures of 4-tetradecanoyl-2,6-piperazinedione, (a) and 3-benzylhexahydropyrrolo(1,2-a)pyrazine-1,4-dione (b) as selected ligand molecules


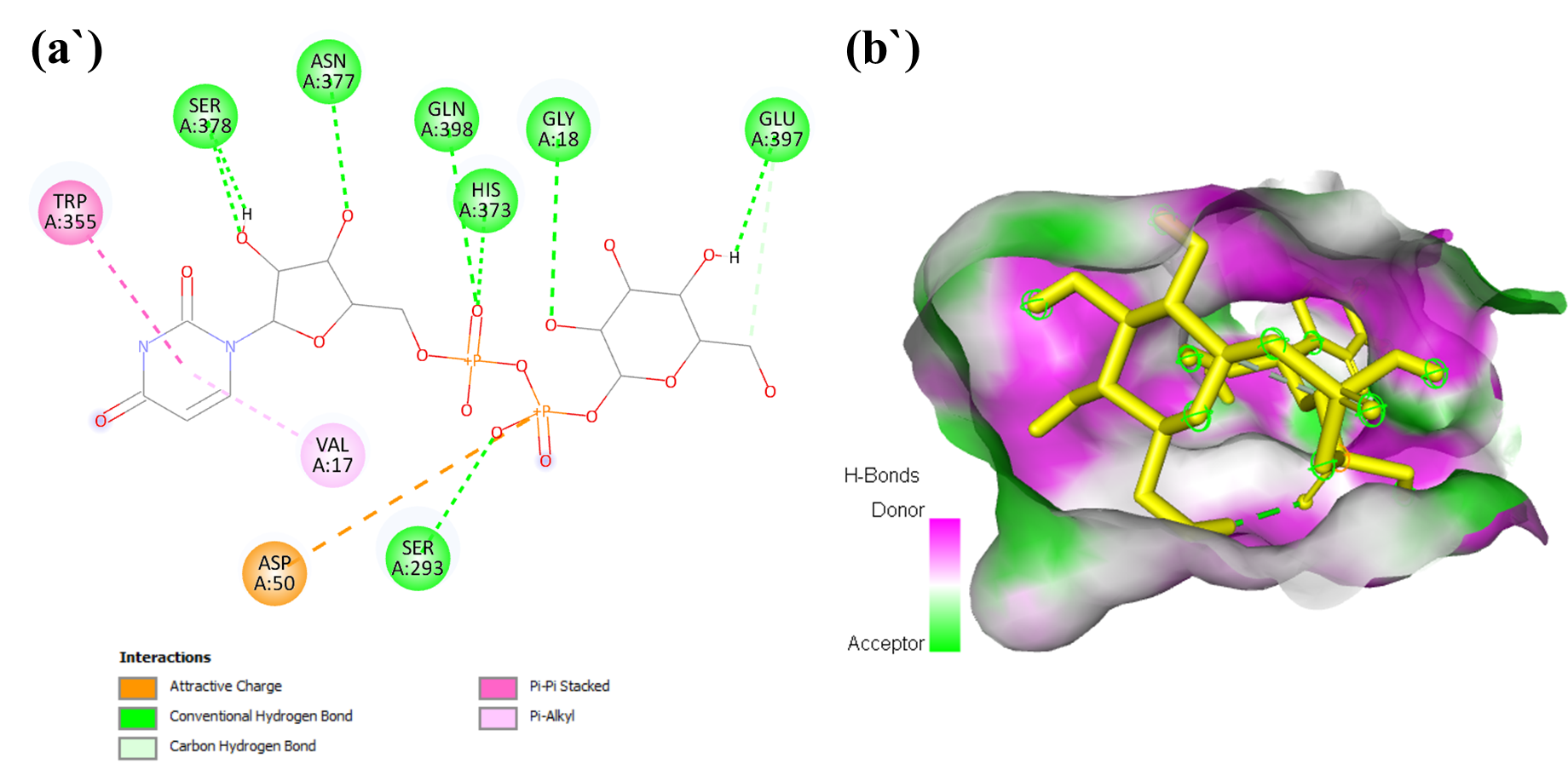


**S3 Fig.** 2D and 3D molecular interaction of uridine-5-diphosphate with target protein residues after re-docking.


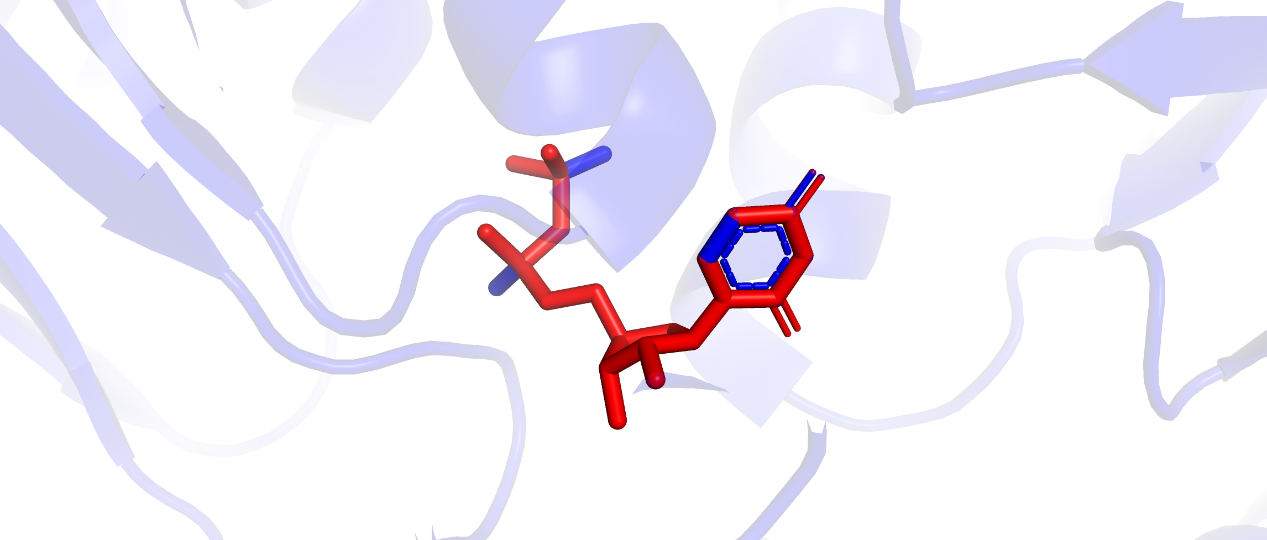


**S4 Fig.**  Superimposition of the re-docked UDP (Red) with the co-crystallized UDP (Blue) within the binding pocket of UGT706F8 (PDB: 7Q3S). The RMSD of 0.9 Å (heavy atoms) between the two poses validates the reproducibility of the docking protocol.


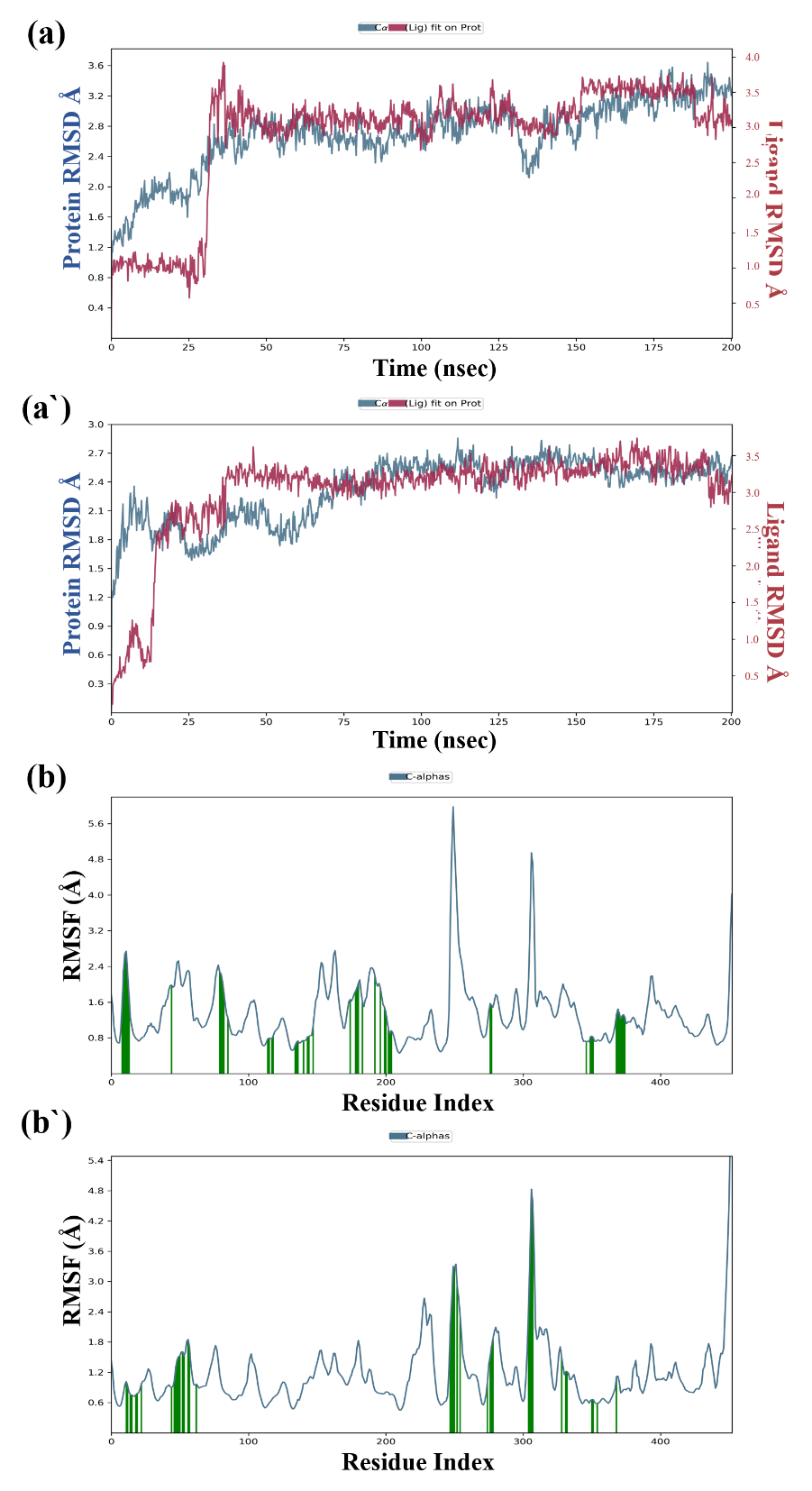


**S5 Fig.** RMSD and residues-wise RMSF values of 4-tetradecanoyl-2,6-piperazinedione-protein complex (a-a`) and 3-benzylhexahydropyrrolo(1,2-a)pyrazine-1,4-dione-protein complex (b-b`) over 200 ns MD simulation.


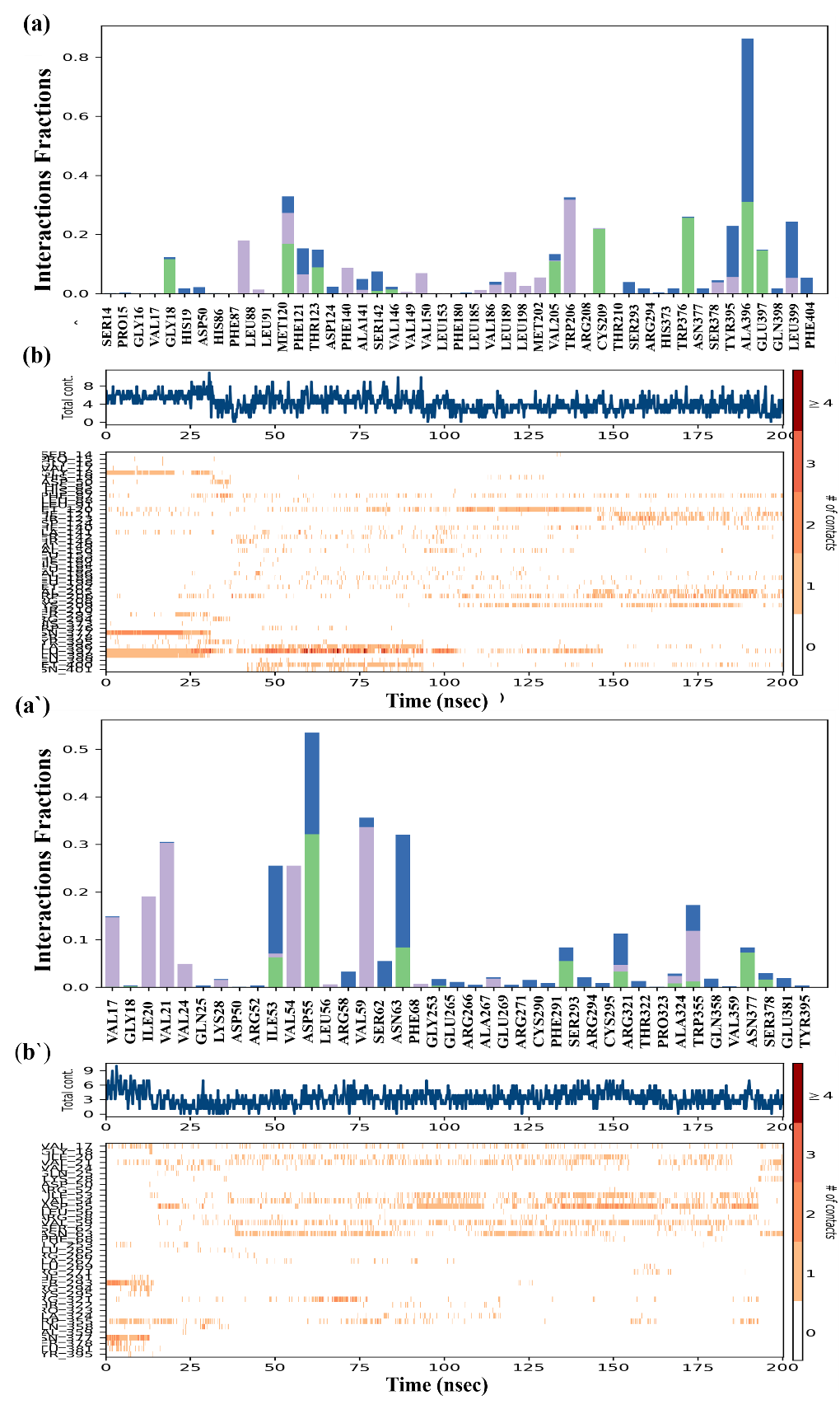


**S6 Fig.** Protein-ligand interaction histograms and a timeline representation of 4-tetradecanoyl-2,6-piperazinedione-protein complex (a-a`) and 3-benzylhexahydropyrrolo(1,2-a)pyrazine-1,4-dione-protein complex (b-b`) showing hydrogen bonding, water bridge, and hydrophobic contacts.

**
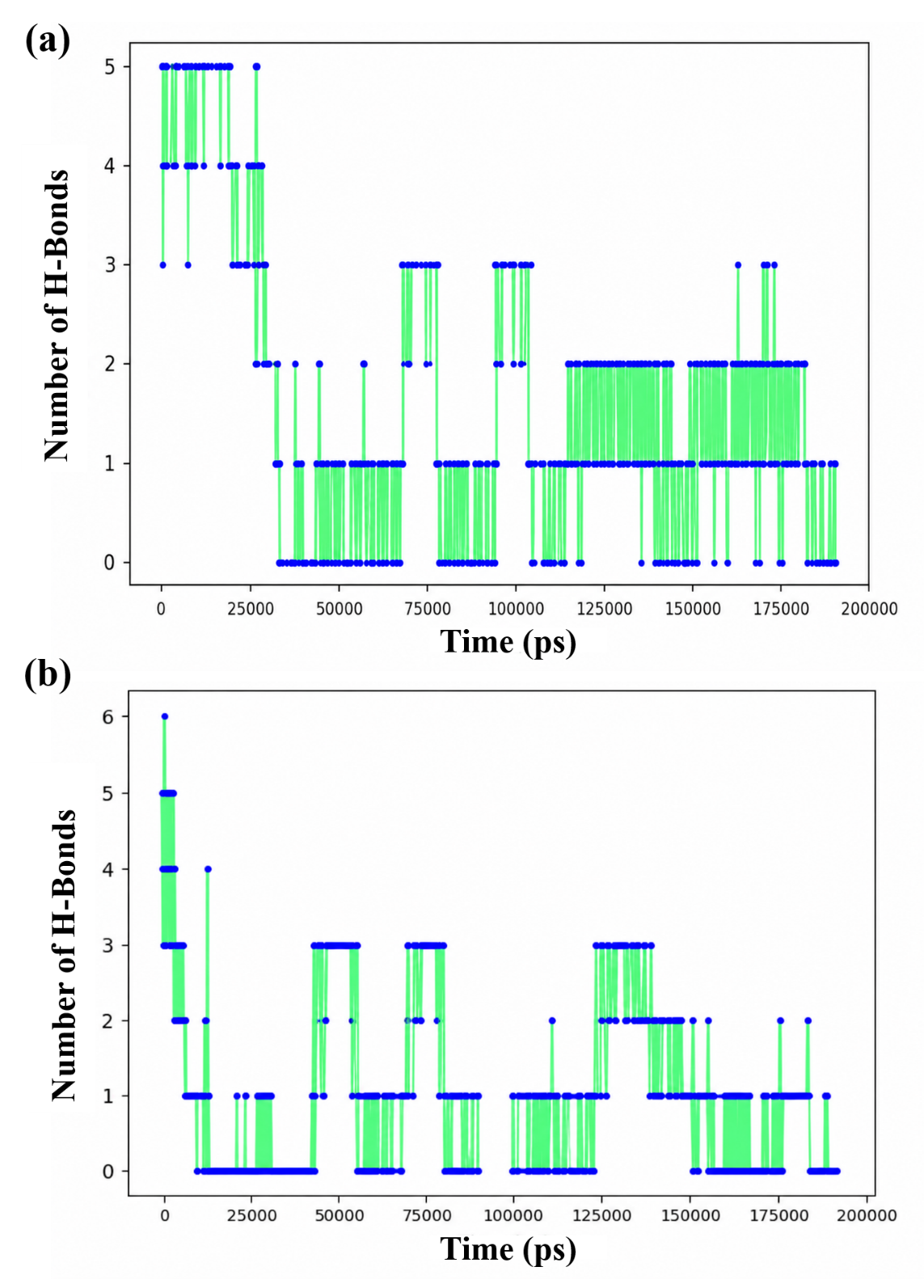
**

**S7 Fig.** H-bond occupancy analysis of 4-tetradecanoyl-2,6-piperazinedione-protein complex (a) and 3-benzylhexahydropyrrolo(1,2-a)pyrazine-1,4-dione-protein complex (b) displaying hydrogen bonding over the course of a 200 ns simulation.


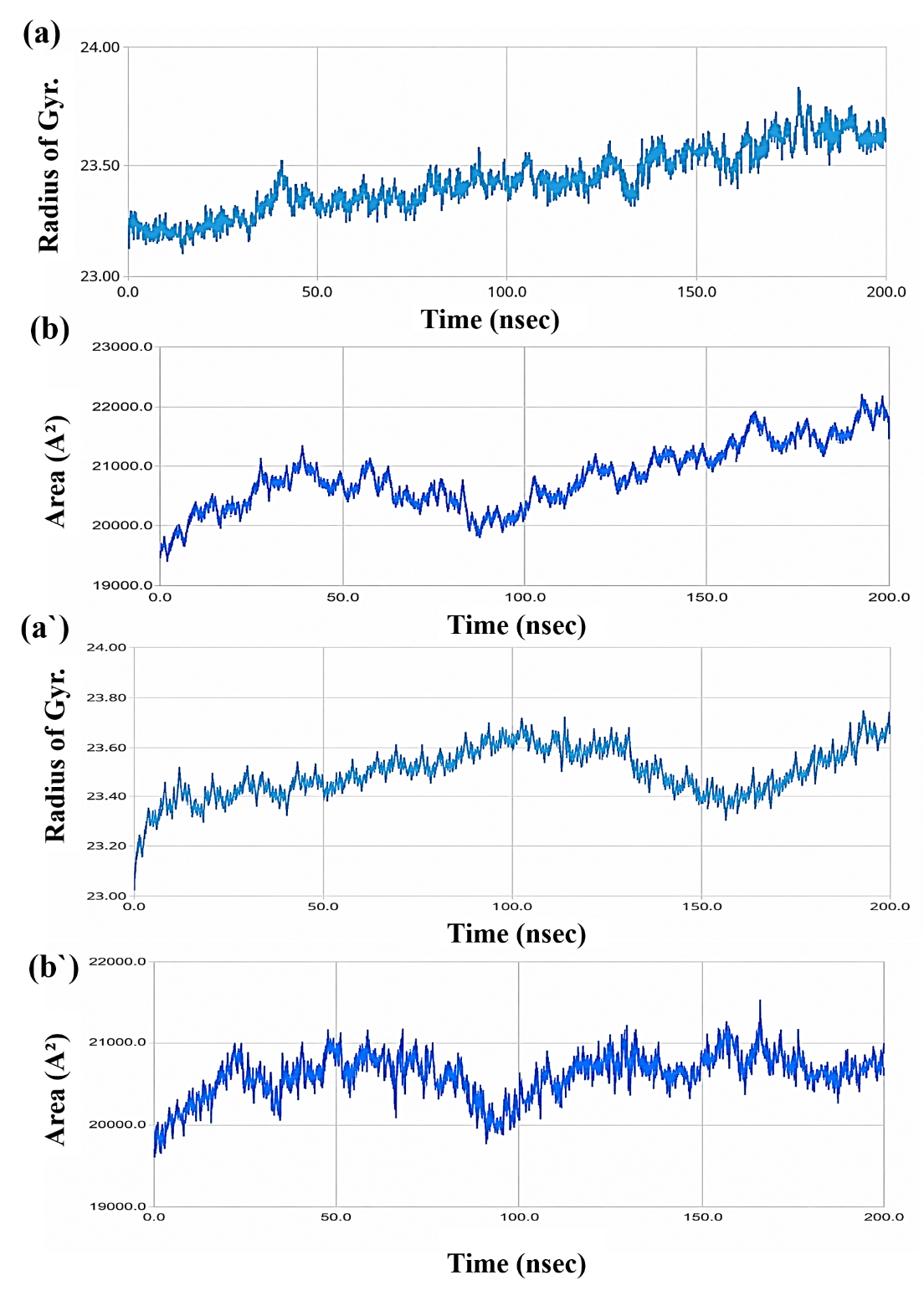


**S8 Fig.** Rg analysis and SASA profiles of 4-tetradecanoyl-2,6-piperazinedione-protein complex (a-a`) and 3-benzylhexahydropyrrolo(1,2-a)pyrazine-1,4-dione-protein complex (b-b`) showing minor conformational changes in UGT706F8 protein upon ligand binding over the course of a 200 ns simulation.


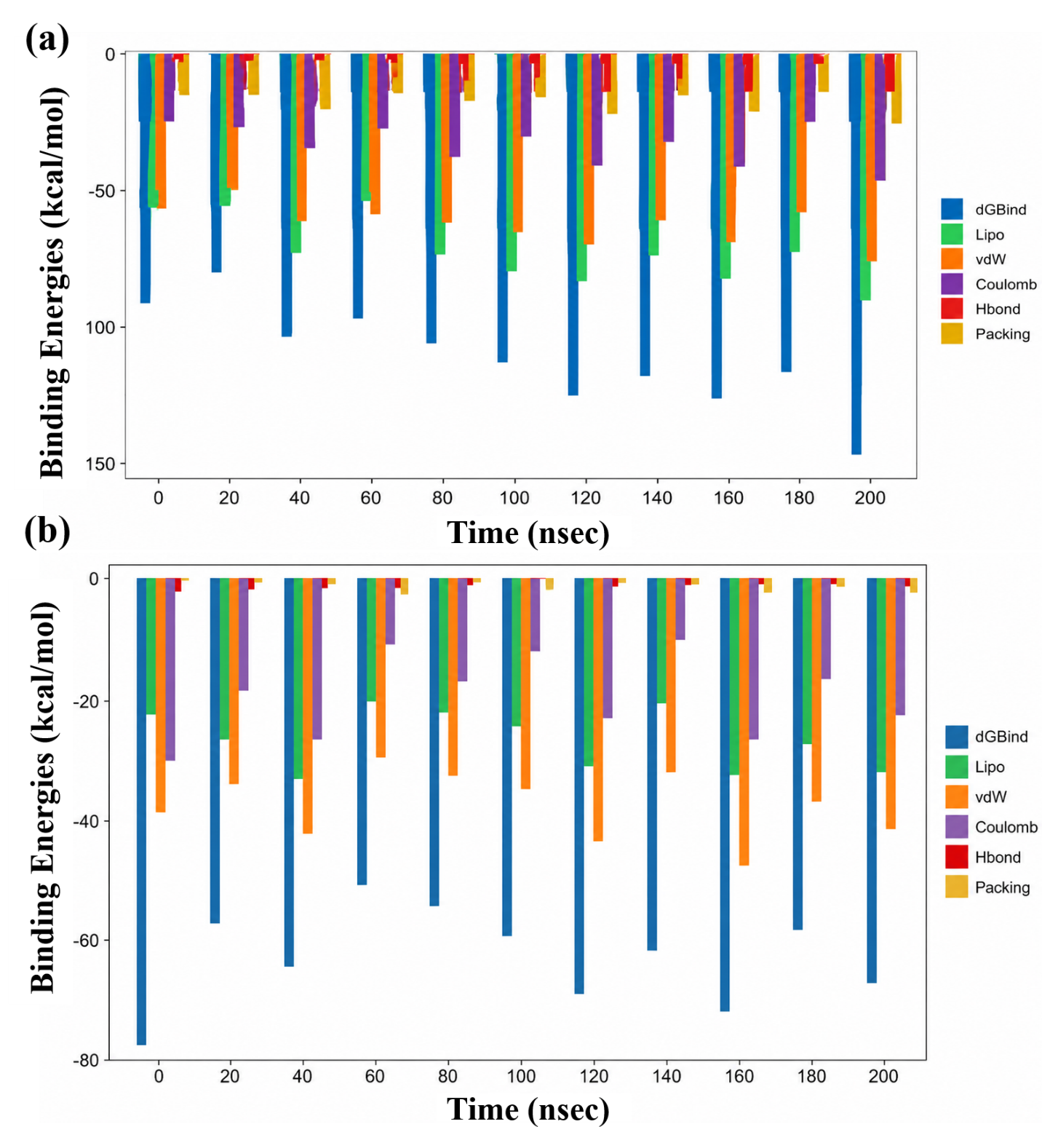


**S9 Fig.** MM-GBSA analysis was conducted to predict binding free energies and energy components of 4-tetradecanoyl-2,6-piperazinedione-protein (a) and 3-benzylhexahydropyrrolo(1,2-a)pyrazine-1,4-dione-protein (b) complexes.


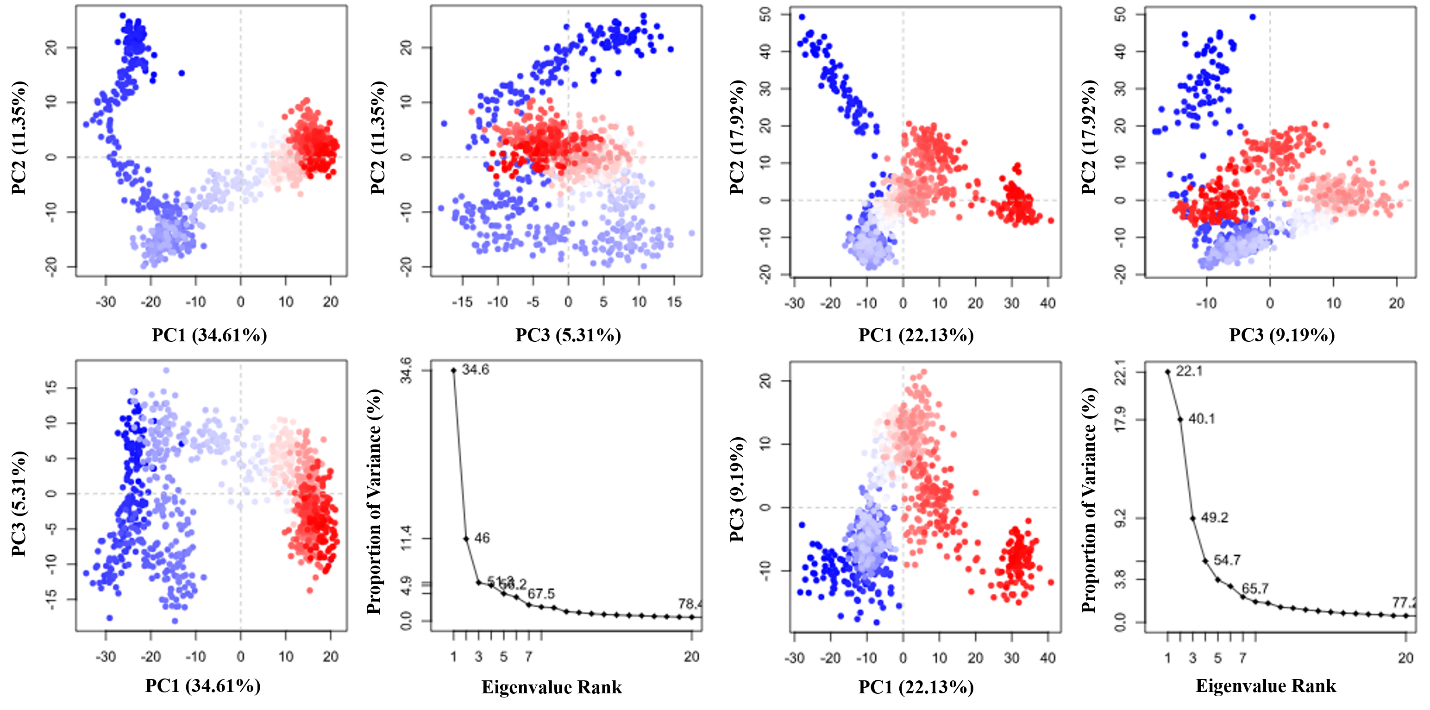


**S10 Fig.** 2D projection of Principal Component analysis of 4-tetradecanoyl-2,6-piperazinedione-protein complex (a) and 3-benzylhexahydropyrrolo(1,2-a)pyrazine-1,4-dione-protein complex (b). Each panel displays PC1-PC2, PC1-PC3, and PC2-PC3 projections, where the colors of the dots (red = final, blue = initial, and white = intermediate) indicate conformations by simulation time.


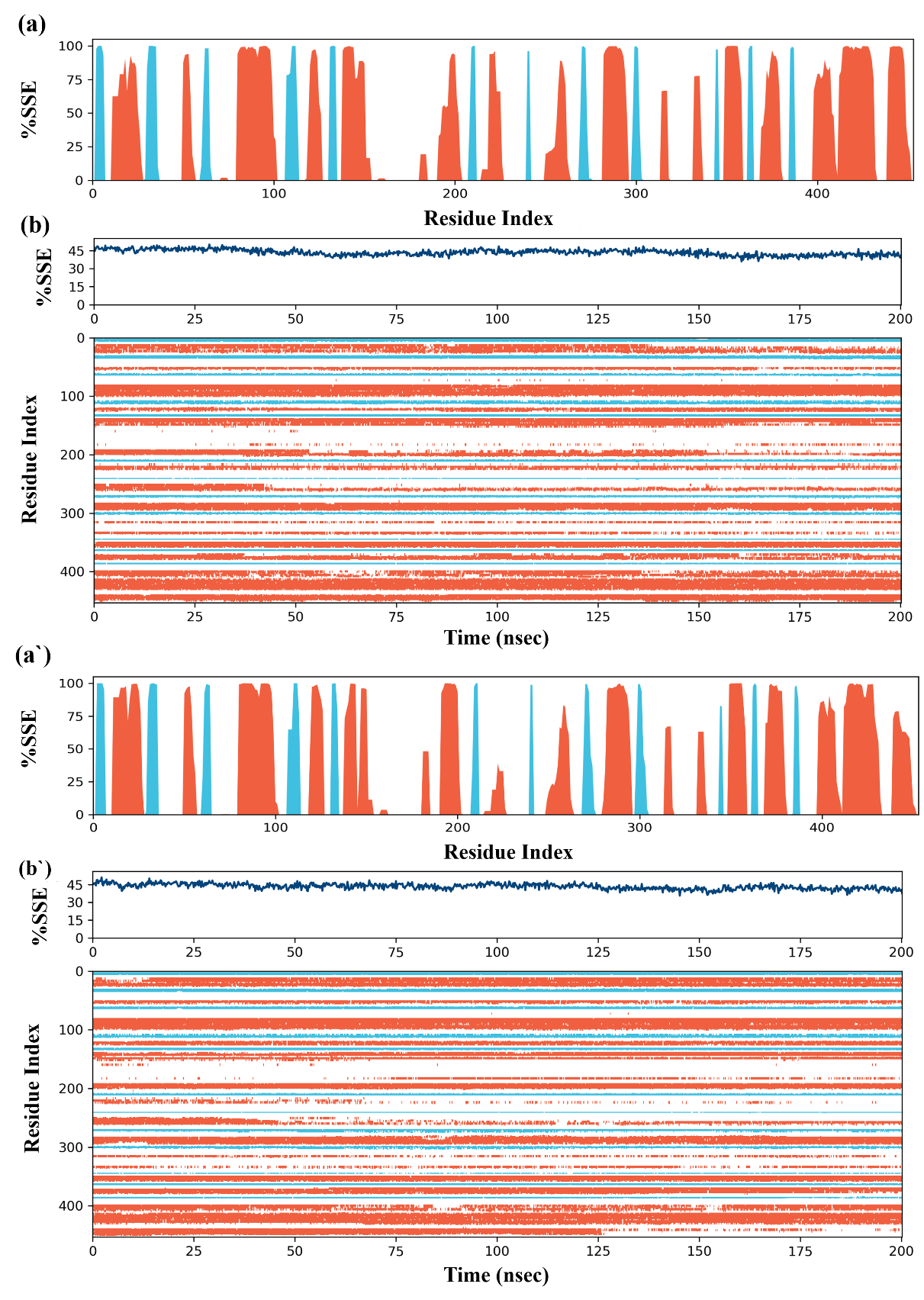


**S11 Fig.** Dictionary of Secondary Structure of Proteins (DSSP) analysis of 4-tetradecanoyl-2,6-piperazinedione-protein (a) and 3-benzylhexahydropyrrolo(1,2-a)pyrazine-1,4-dione-protein (b) complexes, revealing the evolution of secondary structural elements over the simulation period.


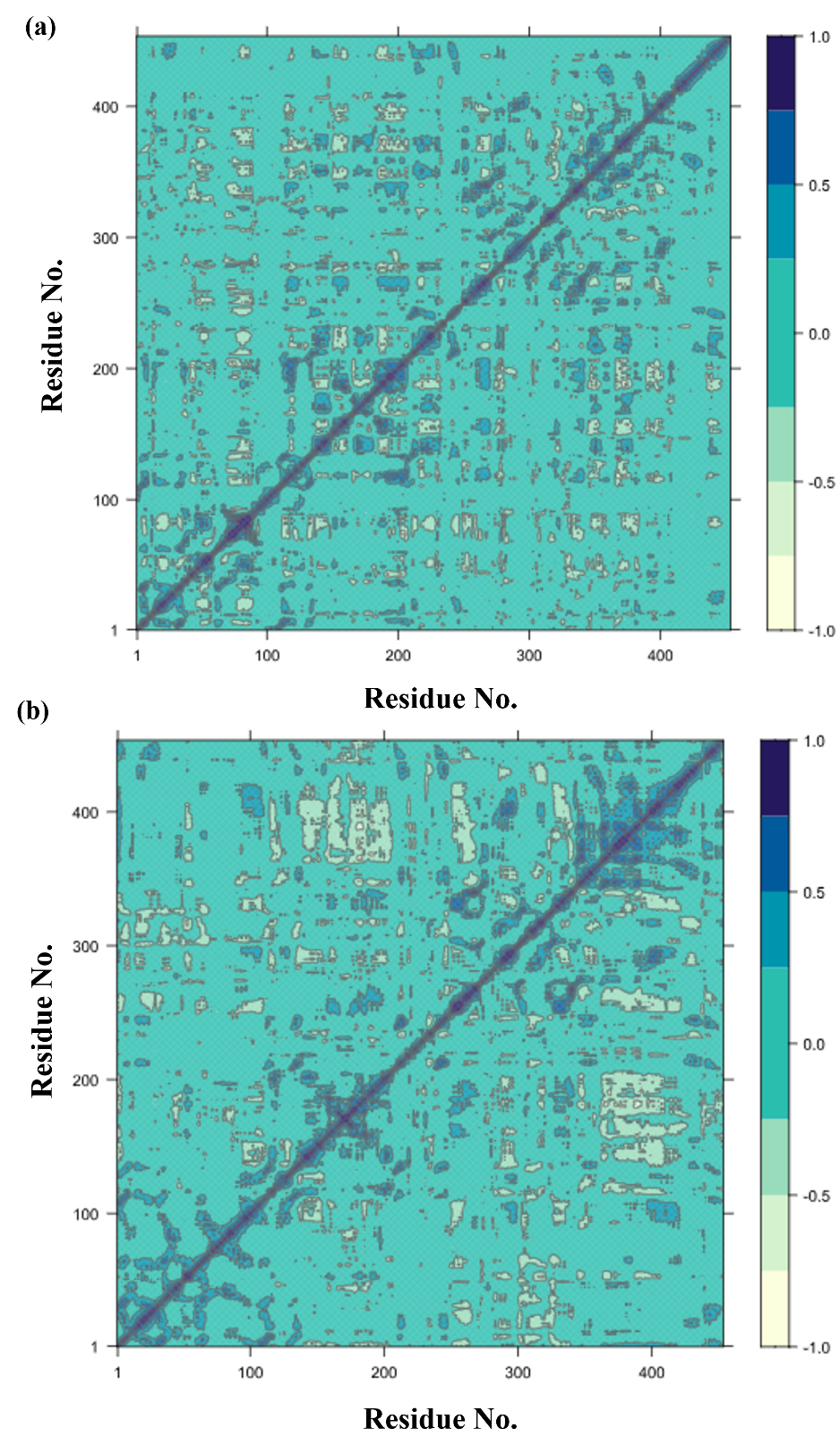


**S12 Fig.** **Dynamic Cross-Correlation Matrix (DCCM)** analysis of 4-tetradecanoyl-2,6-piperazinedione-protein (a) and 3-benzylhexahydropyrrolo(1,2-a)pyrazine-1,4-dione-protein (b) complexes, displaying the correlated (positive) and anti-correlated (negative) residue motion during simulation.
